# Supplementary material for: Understanding the mechanisms of a combined physical and psychological intervention for people with neurogenic claudication: protocol for a causal mediation analysis of the BOOST trial
Source: BMJ Open. 2020 Sep 2;10(9):e037121. doi: 10.1136/bmjopen-2020-037121 (PMC7470505; doi:10.1136/bmjopen-2020-037121)
Supplement: Author's manuscript [file bmjopen-2020-037121.draft_revisions.pdf]

BMJ Open is committed to open peer review. As part of this commitment we make the peer review history of every article we publish publicly available.

When an article is published we post the peer reviewers' comments and the authors' responses online. We also post the versions of the paper that were used during peer review. These are the versions that the peer review comments apply to.

The versions of the paper that follow are the versions that were submitted during the peer review process. They are not the versions of record or the final published versions. They should not be cited or distributed as the published version of this manuscript.

BMJ Open is an open access journal and the full, final, typeset and author-corrected version of record of the manuscript is available on our site with no access controls, subscription charges or pay-per-view fees (http://bmjopen.bmj.com).

If you have any questions on BMJ Open's open peer review process please email info.bmjopen@bmj.com

BMJ Open

Understanding the mechanisms of a combined physical and psychological intervention for people with neurogenic claudication causes changes in pain and disability: Protocol for a causal mediation analysis of the BOOST Trial

Journal:	*BMJ Open*
Manuscript ID	bmjopen-2020-037121
Article Type:	Protocol
Date Submitted by the Author:	19-Jan-2020

Complete List of Authors:	Comer, Christine; Leeds Community Healthcare NHS Trust, Musculoskeletal and Rehabilitation Services; University of Leeds Faculty of Medicine and Health, Leeds Institute of Rheumatic and Musculoskeletal Medicine<br>Lee, Hopin; Hunter New England Population Health; University of Oxford Nuffield Department of Orthopaedics Rheumatology and Musculoskeletal Sciences<br>Williamson, Esther; Nuffield Department of Orthopaedics, Rheumatology and Musculoskeletal Sciences, University of Oxford, United Kingdom. , Centre for Rehabilitation Research in Oxford<br>Lamb, Sarah; University of Exeter, College of Medicine and Health; University of Oxford, NDORMS

Keywords:	Spine < ORTHOPAEDIC & TRAUMA SURGERY, GERIATRIC MEDICINE, REHABILITATION MEDICINE

SCHOLARONE™
Manuscripts

I, the Submitting Author has the right to grant and does grant on behalf of all authors of the Work (as defined in the below author licence), an exclusive licence and/or a non-exclusive licence for contributions from authors who are: i) UK Crown employees; ii) where BMJ has agreed a CC-BY licence shall apply, and/or iii) in accordance with the terms applicable for US Federal Government officers or employees acting as part of their official duties; on a worldwide, perpetual, irrevocable, royalty-free basis to BMJ Publishing Group Ltd ("BMJ") its licensees and where the relevant Journal is co-owned by BMJ to the co-owners of the Journal, to publish the Work in this journal and any other BMJ products and to exploit all rights, as set out in our licence.

The Submitting Author accepts and understands that any supply made under these terms is made by BMJ to the Submitting Author unless you are acting as an employee on behalf of your employer or a postgraduate student of an affiliated institution which is paying any applicable article publishing charge ("APC") for Open Access articles. Where the Submitting Author wishes to make the Work available on an Open Access basis (and intends to pay the relevant APC), the terms of reuse of such Open Access shall be governed by a Creative Commons licence – details of these licences and which Creative Commons licence will apply to this Work are set out in our licence referred to above.

Other than as permitted in any relevant BMJ Author's Self Archiving Policies, I confirm this Work has not been accepted for publication elsewhere, is not being considered for publication elsewhere and does not duplicate material already published. I confirm all authors consent to publication of this Work and authorise the granting of this licence.

For peer review only

Understanding the mechanisms of a combined physical and psychological intervention for people with neurogenic claudication causes changes in pain and disability: Protocol for a causal mediation analysis of the BOOST Trial

Christine Comer [1,2,] Hopin Lee [3,4] Esther Williamson [3] and Sarah Lamb [3,5]

1 Leeds Community Healthcare Musculoskeletal and Rehabilitation Services, Leeds Community Healthcare NHS Trust, Stockdale House, Victoria Road, Leeds UK

2 Leeds Institute of Rheumatic and Musculoskeletal Medicine, University of Leeds, Leeds, UK

3 Centre for Rehabilitation Research, Nuffield Department of Orthopaedics, Rheumatology and Musculoskeletal Sciences, University of Oxford, Oxford, UK

4 School of Medicine and Public Health, University of Newcastle, New South Wales, Australia

5 University of Exeter Medical School, Institute of Health Research, Devon, UK

Correspondence to Christine Comer, c.comer@leeds.ac.uk, +44 (0)113 3921521

Authors' contributions: all authors were involved in the design and write-up of this protocol.

Funding statement: Christine Comer is funded by a National Institute for Health Research (NIHR) Clinical Lectureship ICA-CL-2017-03-015 for this research project. This publication presents independent research. The views expressed are those of the author(s) and not necessarily those of the NHS, the NIHR or the Department of Health and Social Care.

Competing interests statement: None of the authors have competing interests to declare

Word count 3,131

Keywords

Mediation, Causality, Spinal Stenosis, Behaviour and behaviour mechanisms, Exercise, Rehabilitation

For peer review only - http://bmjopen.bmj.com/site/about/guidelines.xhtml

ABSTRACT

Introduction

Conservative treatments such as exercise are recommended for the management of people with neurogenic claudication from spinal stenosis [1-3]. However, the effectiveness and mechanisms of effect are unknown. This protocol outlines an a-priori plan for a secondary analysis of a multi-centre randomised controlled trial of a physiotherapist-delivered, combined physical and psychological intervention (Better Outcomes for Older people with Spinal Trouble (BOOST) programme).

Methods and analyses

We will use causal mediation analysis to estimate the mechanistic effects of the BOOST programme on the primary outcome of disability (measured by the Oswestry Disability Index). The primary mechanism of interest is walking capacity, and secondary mediators include fear avoidance behaviour, walking self-efficacy, physical function, physical activity and/or symptom severity. All mediators will be measured at 6 months and the outcome will be measured at 12 months from randomisation. Patient characteristics and possible confounders of the mediator-outcome effect will be measured at baseline. Sensitivity analyses will be conducted to evaluate the robustness of the estimated effects to varying levels of residual confounding.

Ethics and Dissemination

Ethical approval was given on the 03 March 2016 (National Research Ethics Committee number: 16/LO/0349). The results of this analysis will be disseminated in peer-reviewed journals and at relevant scientific conferences.

Trial Registration number ISRCTN12698674

Strengths and Limitations of the Study

- Causal mediation analysis of clinical trials can explain how an effective intervention works, or why an ineffective intervention does not work.
- We propose exploratory analysis of several potential causal pathways based on theoretical mechanisms of action and we acknowledge the risks associated with this multiple testing.
- The overall objective of this study is to identify the mechanisms that underlie the effect of the BOOST programme.
- Understanding these treatment mechanisms will provide valuable information to guide future optimisation or implementation.

For peer review only - http://bmjopen.bmj.com/site/about/guidelines.xhtml

INTRODUCTION

Neurogenic claudication (NC) is a leading cause of disability and lost independence in older adults [4-6]. NC is the term used to describe the typical symptoms associated with lumbar spinal stenosis (LSS) [7]. These symptoms are provoked by walking, and include pain, aching, paraesthesia and fatigue which radiate from the spine into the buttocks and legs [8]. NC often leads to reduced mobility [9] contributing to disability, frailty and falls [10-15], increased risks of comorbidity, social isolation and loss of independence [16, 17]. The symptoms are thought to arise because of reduced space and subsequent compromise of the neural and vascular elements in the lumbar spine [7] resulting from degenerative changes in the aging spine [18].

LSS has become the most common reason for spinal surgery in people over 65 years of age [19]. Because of the risks, costs and unclear benefits associated with surgical treatment [19-21], it is widely accepted that non-surgical treatment should be first line care [22]. Advice, exercise and self-management strategies are often used in clinical practice [23, 24] but there is limited robust evidence to demonstrate their efficacy and effectiveness [20].

The BOOST trial is a large multicentre RCT testing the effectiveness of a 12-week combined physical and psychological intervention delivered by physiotherapists in a group setting. The primary focus of this randomised trial is to estimate the effect of the BOOST intervention on patient-reported disability. There is, however, growing recognition of the importance of evaluating hypothesised treatment mechanisms in randomised trials [25]. Doing so can explain how an effective intervention works, or why an ineffective intervention does not work.

OBJECTIVES

We present an a priori protocol for a secondary analysis of the BOOST trial to evaluate the effects on the primary mediator of a change in walking capacity, and to explore alternative mediators of changes in fear avoidance behaviour, walking self-efficacy, physical function, physical activity, and symptom severity. The aim of this planned analysis is to contribute to our understanding of treatment mechanisms and provide valuable information to guide future intervention development and implementation.

METHODS AND ANALYSES

Design

This study is a causal mediation analysis of the National Institute of Health Research (NIHR) funded BOOST trial. The BOOST trial [ISRCTN12698674] is a high-quality multi-centred randomised controlled trial. It aims to evaluate the clinical- and cost-effectiveness of a physiotherapist-delivered, combined physical and psychological intervention for community-dwelling older adults with symptoms of NC, compared to

For peer review only - http://bmjopen.bmj.com/site/about/guidelines.xhtml

a control treatment of best practice (standardised advice and education). BOOST trial details are published separately in the trial protocol [26].

Participants

438 participants with symptoms of NC from across 15 primary- and secondary- care centres in the UK have been randomised to the trial intervention or control treatment at a 2:1 ratio. We recruited participants over the age of 65 years from spinal clinics within primary and secondary care centres (n=394) and via a primary care survey of community dwelling older adults (n=44). They were eligible for the study if they presented with symptoms consistent with NC (back and/or leg pain or symptoms aggravated by standing or walking and eased by sitting or bending) and were able to participate safely in the group rehabilitation programme.

BOOST trial Intervention

A detailed description of the BOOST intervention and its development have been published elsewhere [27]. In summary, the physical component of the BOOST intervention comprises a 60-minute exercise programme of exercises for strength, balance and flexibility, and a walking circuit. The level of exercise difficulty is individually tailored, and is progressed over the 12-week programme by increasing the number of repetitions, sets, and increasing weights and/ or speed. The psychological component consists of a series of 30-minute discussion sessions. Early sessions are aimed at facilitating self-efficacy and safety, including addressing unhelpful pain behaviour and fear-related avoidance of movement; providing simple concepts of exercise physiology relevant to improving mobility; and introducing basic behavioural techniques of pacing and graded activity. Subsequent sessions use peer-support to encourage exercise adherence, identify facilitators and barriers, and reinforce long-term exercise adherence through peer discussion about exercise confidence and motivation, access to local activities, and advice on coping with acute symptom flare-ups. Over the 12-week supervised programme, session frequency reduces from twice weekly to once weekly and then to once fortnightly. Concurrently, home exercise programmes are introduced with the aim of encouraging participants to increase their level of independent, unsupervised exercise and physical activity at home.

We developed the intervention collaboratively with clinical experts and patients to address some of the unique challenges of managing back problems in older people. These include age-related physical changes of reduced muscle strength and fitness, in addition to psychological factors such as depression [28] and unhelpful behaviours and beliefs about aging and exercise which are known to negatively impact on outcomes and act as barriers to accessing and engaging with treatment [29-32].

Control intervention

The control intervention consists of a one-hour individual session with a physiotherapist for assessment, advice and education about NC and being physically active, advice on exercises including flexion-based exercises, use of medications and when to seek more advice. The advice and education are reinforced in a leaflet and a further two review appointments are available if required.

For peer review only - http://bmjopen.bmj.com/site/about/guidelines.xhtml

Outcomes

The Oswestry Disability Index (ODI) [33] is the primary outcome measure for the BOOST Trial. The ODI (also known as the Oswestry Low Back Pain Disability Questionnaire) is the most widely used measure in low back pain research and is considered the 'gold standard' of low back functional outcome tools. It is commonly used to provide a patient reported measure of walking limitation and other functional limitations in Lumbar spinal stenosis trials, alongside objective measures of walking ability [34].

The ODI comprises a series of questions about how pain impacts on the patient's ability to carry out a range of daily functions. For each question, possible answers are scored on a scale of 0 to 5. These scores are summed and expressed as a percentage of the maximum possible score for disability, with higher scores representing worse disability. In the BOOST trial, ODI scores are measured for each participant at baseline, and at 6 and 12 months after randomisation.

Important secondary outcome measures for the BOOST trial focus on walking capacity, and
on fear avoidance behaviour, walking self-efficacy, physical function, physical activity and
symptom severity: The six-minute walk test provides an objective measure of walking capacity and entails a timed measure of walking distance which is easily performed in the clinical setting [35]. The Short Physical Performance Battery (SPPB) provides an objective measure of physical performance which incorporates a balance test, a functional lower limb strength test of 5 timed chair stands, and a timed walking test over 8 feet. This is similarly easy to complete in a clinic [36]. Outcome questionnaires completed by participants provide subjective evaluation of relevant constructs. These include physical activity measured by a modified version of the Rapid Assessment Disuse Index (RADI) [37]; severity of stenotic symptoms measured by the Swiss Spinal Stenosis Questionnaire (SSS) symptom severity subscale [38]; walking self-efficacy measured by a single item from the Modified Gait Efficacy Scale (GES) [39]; and fear of movement measured by the physical activity subscale of the Fear Avoidance Beliefs Questionnaire (FABQ) [40]. These outcomes are all measured at baseline, and at 6 and 12 months following randomisation.

Causal Mediation Analysis - Rationale

We will test the mechanisms of the BOOST intervention for patients with NC by estimating the extent to which different potential mediators explain the effect of the BOOST intervention on participants' ODI outcome scores. This will be achieved by deconstructing the total effect of the BOOST treatment into indirect and direct effects. The indirect effect is the path through which the intervention affects the outcome via a selected mediator. The direct effect is the remaining total effect that is not encompassed by the indirect effect.

Walking capacity has been selected as the primary mediator for our causal mediation analysis model. Walking limitation is a hallmark of disability in LSS [41] and a serious concern for people with LSS who often seek healthcare because of reduced mobility [42-44]. The BOOST intervention incorporates both physical and

For peer review only - http://bmjopen.bmj.com/site/about/guidelines.xhtml

cognitive behavioural components aimed at reducing disability, and increased walking is a key target.

The BOOST intervention is underpinned by a strong theoretical premise for addressing age-related decline in mobility. Exercise components of the intervention aim to improve physical function by targeting reduced muscle strength, balance and sensory impairments [45-54]. Whilst cognitive behavioural approaches have not been well investigated in people with LSS [55], this is an important component of the BOOST intervention based on evidence that addressing unhelpful beliefs and behaviours can mediate changes in both disability and symptom severity in low back pain populations [34, 56-60]. In addition, self-efficacy is likely to play a role in mediating disability, physical function and activity in older adults with chronic musculoskeletal conditions [61-63]. Alternative mediators that we will include in our causal mediation models therefore include physical function (SPPB), physical activity (RADI), fear avoidance behaviour (FABQ), and walking self-efficacy (GES).

Our final alternative mediator is symptom severity (SSS). Little is known about how exercise affects NC symptom severity [64]. In theory, better muscular strength, control and endurance, and increased flexibility are likely not only to enhance physical ability [65, 66], but also to reduce pain by altering the loading of the musculoskeletal system and changing central pain processes and immune system responses [67-69]. Neuroischaemic lower limb symptoms such as paraesthesia, numbness, sensory and balance changes might also be improved through reduced nerve root ischaemia and venous pooling as a result of exercise [7, 70].

To understand the mechanisms of the BOOST intervention, we plan to test our primary hypothesis that changes in disability will be mediated primarily by changes in walking capacity, and that various mechanisms might explain these changes in walking capacity: We hypothesise that the progressive exercise component of the BOOST intervention will exert its effect on walking capacity via initial changes in physical function and physical activity levels during treatment. We further hypothesise that the cognitive behavioural components of the intervention will exert effects on walking capacity through reductions in fear avoidance behaviour and increased self-efficacy. There is also the potential for both the exercise and the cognitive behavioural components of the intervention to reduce symptom severity, resulting in increased walking capacity.

Our secondary hypothesis is that the causal effects of the BOOST intervention on disability will be explained through changes in fear avoidance behaviour, self-efficacy, physical function, physical activity and/or symptom severity (SSS) without exerting any effects on walking capacity.

If the BOOST intervention is found to be effective, causal mediation analysis will explain how the intervention works. Conversely, if the intervention is not found to be effective, causal mediation analysis will identify where the hypothesised mechanism broke down [71].

For peer review only - http://bmjopen.bmj.com/site/about/guidelines.xhtml

Causal Mediation analysis - models

Table 1 provides an overview of the planned mediation models contingent on whether or not the BOOST intervention is effective:

► If the intervention is effective, our primary objective is to estimate the extent to which changes in walking capacity mediate this effect.

Our secondary objective will be to further refine this model via five serial multiple mediator paths (changes in fear avoidance behaviour, walking self-efficacy, physical function, physical activity, and symptom severity) that may subsequently cause changes in walking capacity.
The serial multiple mediator models accounting for potential confounders are conceptualised in path diagrams called Directed Acyclic Graphics (DAGs) to aid understanding of potential complex causal relationships (Figure 2).

► If the intervention is not effective, our primary objective is to determine where the causal paths break down. All potential mediators (walking capacity, fear avoidance, self-efficacy, physical function, physical activity and symptom severity) will be evaluated.

If alternative pathways of mediation emerge during the analysis of the trial and associated qualitative data these will also be investigated. The publication of this protocol does not preclude other analyses if indicated.

Statistical Analysis

We will use a model-based inference approach for causal mediation analysis [72]. All analyses will be conducted in R (The R Foundation for Statistical Computing) using the "*mediation*" package [73].

We will construct single mediator models for the ODI outcome. For each single mediator model (primary mediator model of walking capacity, and secondary mediator models of fear avoidance, self-efficacy, physical function, physical activity and symptom severity), we will estimate the intervention-mediator effect, the mediator-outcome effect, the average causal mediation effect (ACME), average direct effect (ADE) and the average total effect (ATE). We will also estimate the proportion mediated in each single mediator model. The ACME is the average intervention effect through the mediator; ADE is the average intervention effect that works through all other mechanism excluding the selected mediator; and the ATE is the average effect of the intervention on the outcome. The ATE is the sum of the ACME and ADE on the additive scale. The proportion mediated is the fraction of the ATE that is explained by the ACME.

We will fit two regression models: the mediator model, and the outcome model. The mediator model will use linear regression with treatment allocation as the independent variable, and the mediator as the dependent variable, with the baseline level of the mediator as a covariate. The outcome model for disability will also use linear regression. Each outcome model will be constructed with the mediator as the independent variable, the outcome as the dependent variable, and treatment allocation, baseline values of the meditator and outcome variable in addition to the

For peer review only

set of observed pre-treatment confounders as covariates. Because the mediators are not randomised, it is possible for the mediator-outcome effects to be confounded. To address this problem, we will adjust for the effects of pre-treatment confounders based on theorised causal relationships with the mediators and outcome variables from expert clinical knowledge and available evidence. These potential confounders include age, BMI, sex, number of comorbidities, in addition to baseline measures of disability, physical function, walking capacity, physical activity, symptom severity and fear-avoidance behaviour, and baseline frailty (frailty index/ grip strength); use of walking aids; general pain (Nordic pain Q); exercise self-efficacy; and attitudes to aging. We will also conduct a sensitivity analysis to determine the robustness of the estimated ACME and ADE to possible unmeasured confounding [74]. We will also include an interaction term (treatment allocation X mediator) in the outcome models to allow for an intervention-mediator interaction effect on the outcome and to increase model flexibility [75].

Using mediator and outcome regression models, we will simulate potential values of the mediator for each participant under each level of the intervention; then simulate potential outcome values for each participant under all combinations of the intervention and simulated mediator values. From these observed and simulated potential values of the mediator and outcome, we will calculate point estimates for the ACME, ADE, and TE and their 95% confidence intervals using 1000 bootstrapped simulations.

For multiple mediator models, we will use an expanded framework. [76] Multiple mediator models will only be constructed if the alternative mediator (fear avoidance, self-efficacy, physical function, physical activity and symptom severity) and primary mediator (walking capacity) are associated [76]. We will use the *multimed* function from the *mediation* package to estimate the ACME and ADE, and the sensitivity parameters.

We will assess the proportion of missing mediator and outcome data. We will conduct all analyses on complete cases if the proportion of missing data is <15%. If missing data exceeds 15% we will impute missing data through multiple imputation by chain equations using the "mice" package [77] in R.

To facilitate clinical interpretation, we will calculate how much change in a given mediator would equate to a minimally clinically important difference (MCID) of disability (5 point change in ODI score [78]) by using the regression coefficients from the outcome model, we will divide the MCID by the mediator coefficient and 95% Confidence Interval from the outcome regression model.

DISCUSSION

We do not currently know whether non-surgical interventions are effective in reducing disability reported by older people with NC symptoms. The BOOST trial will provide important high-quality evidence about the effectiveness of an intervention incorporating components to address both physical and psychological impairments. The targets of this comprehensive intervention include walking capacity, physical function, physical activity, self-efficacy, unhelpful beliefs about activity, and symptom

For peer review only - http://bmjopen.bmj.com/site/about/guidelines.xhtml

severity. We cannot assume, however, that if the intervention improves any of these variables the participants will experience benefits in patient-reported outcomes such as the ODI.

Mediation analysis conducted in parallel to an RCT can address specific questions about the underlying mechanisms of the intervention being tested. This protocol describes the planned causal mediation analysis that we will undertake to evaluate hypothetical mechanisms that underlie the effect of the BOOST rehabilitation programme. This will help us to understand how the BOOST intervention works, or doesn't work.

If the BOOST intervention is found to be effective and this mediation analysis indicates that improvements in ODI scores are mediated by walking capacity, which in turn is mediated by improved self-efficacy, then this provides valuable information for refining the intervention with greater focus on self-efficacy to optimise treatment effectiveness. If, on the other hand, the BOOST trial is not found to be produce a significant improvement in patient reported disability, then the analysis will help us to understand where the hypothesises causal mechanisms of treatment break down. For example, we may find that although the BOOST intervention improves walking capacity, this does not translate into an improvement in ODI score and may therefore not be an appropriate treatment target. The causal analysis will therefore provide valuable information for designing, refining and implementing an effective intervention.

Because this is an exploratory study we have selected several mediators based on our current clinical understanding and evidence for exercise and behavioural treatments. Several potential causal pathways will therefore be explored, and we acknowledge the risks associated with this multiple testing. If alternative pathways of mediation emerge during the analysis of the trial and associated qualitative data, we will investigate these further. The publication of this protocol does not preclude other analyses if indicated. Future studies may be needed to further explore the inter-relationship of the mediators explored in our analyses.

CONCLUSIONS

Clinical trials do not address questions around *how* an intervention affects outcomes, and therefore may not identify the most effective treatment targets for a particular patient population. Mediation analysis of clinical trials can estimate how much the total effect of the treatment can be attributed to pre-specified treatment targets. Improving our understanding of these treatment mechanisms can generate evidence that can be used to tailor and refine treatments and optimise clinical effectiveness. In this study, we will estimate the causal mediation effects of a rehabilitation programme incorporating exercise and behavioural components for patients with NC.

Ethics: Ethical approval was given on the 03 March 2016 (National Research Ethics Committee number: 16/LO/0349).

Authors' contributions: all authors were involved in the design and write-up of this protocol.

For peer review only - http://bmjopen.bmj.com/site/about/guidelines.xhtml

Funding statement: Christine Comer is funded by a National Institute for Health Research (NIHR) Clinical Lectureship ICA-CL-2017-03-015 for this research project. This publication presents independent research. The views expressed are those of the author(s) and not necessarily those of the NHS, the NIHR or the Department of Health and Social Care.

Competing interests statement: None of the authors have competing interests to declare

For peer review only

REFERENCES

1. Delitto, A., et al., *Surgery versus nonsurgical treatment of lumbar spinal stenosis: a randomized trial.* Ann Intern Med, 2015. **162**(7): p. 465-73.
2. Matsuwaka, S.T., B.C.J.C.P.M. Liem, and R. Reports, *The Role of Exercise in Treatment of Lumbar Spinal Stenosis Symptoms.* 2018. **6**(1): p. 36-44.
3. Rousing, R., et al., *Danish national clinical guidelines for surgical and nonsurgical treatment of patients with lumbar spinal stenosis.* Eur Spine J, 2019. **28**(6): p. 1386-1396.
4. Ishimoto, Y., et al., *Prevalence of symptomatic lumbar spinal stenosis and its association with physical performance in a population-based cohort in Japan: the Wakayama Spine Study.* Osteoarthritis Cartilage, 2012. **20**(10): p. 1103-8.
5. Fanuele, J.C., et al., *The impact of spinal problems on the health status of patients: have we underestimated the effect?* Spine (Phila Pa 1976), 2000. **25**(12): p. 1509-14.
6. Winter, C.C., et al., *Walking ability during daily life in patients with osteoarthritis of the knee or the hip and lumbar spinal stenosis: a cross sectional study.* BMC Musculoskelet Disord, 2010. **11**: p. 233.
7. Lurie, J. and C. Tomkins-Lane, *Management of lumbar spinal stenosis.* BMJ, 2016. **352**: p. h6234.
8. Kreiner, D., et al., *Evidence-Based Clinical Guidelines for Multidisciplinary Spine Care.* 2011: p. 43-44.
9. Ammendolia, C., et al., *What interventions improve walking ability in neurogenic claudication with lumbar spinal stenosis? A systematic review.* 2014. **23**(6): p. 1282-1301.
10. Eggermont, L.H., R.H. Shmerling, and S.G. Leveille, *Tender point count, pain, and mobility in the older population: the mobilize Boston study.* The Journal of Pain, 2010. **11**(1): p. 62-70.
11. Koponen, M.P., et al., *Analgesic use and frailty among community-dwelling older people.* Drugs & aging, 2013. **30**(2): p. 129-136.
12. Leveille, S.G., et al., *The pathway from musculoskeletal pain to mobility difficulty in older disabled women.* Pain, 2007. **128**(1-2): p. 69-77.
13. Leveille, S.G., et al., *Chronic musculoskeletal pain and the occurrence of falls in an older population.* Jama, 2009. **302**(20): p. 2214-2221.
14. Makris, U.E., et al., *Restricting Back Pain and Subsequent Mobility Disability in Community-Living Older Persons.* Journal of the American Geriatrics Society, 2014. **62**(11): p. 2142-2147.
15. Shega, J.W., et al., *Persistent pain and frailty: a case for homeostenosis.* Journal of the American Geriatrics Society, 2012. **60**(1): p. 113-117.
16. Fried, L.P., et al., *Frailty in older adults: evidence for a phenotype.* The Journals of Gerontology Series A: Biological Sciences and Medical Sciences, 2001. **56**(3): p. M146-M157.
17. Troutman-Jordan, M. and J. Staples, *Successful aging from the viewpoint of older adults.* Res Theory Nurs Pract, 2014. **28**(1): p. 87-104.
18. Chad, D.A.J.N.c., *Lumbar spinal stenosis.* 2007. **25**(2): p. 407-418.
19. Deyo, R.A., et al., *Trends, major medical complications, and charges associated with surgery for lumbar spinal stenosis in older adults.* JAMA, 2010. **303**(13): p. 1259-65.
20. Zaina, F., et al., *Surgical Versus Nonsurgical Treatment for Lumbar Spinal Stenosis.* Spine, 2016. **41**(14): p. E857-E868.
21. Kovacs, F.M., G. Urrutia, and J.D. Alarcon, *Surgery versus conservative treatment for symptomatic lumbar spinal stenosis: a systematic review of randomized controlled trials.* Spine (Phila Pa 1976), 2011. **36**(20): p. E1335-51.
22. Ammendolia, C., et al., *Nonoperative treatment for lumbar spinal stenosis with neurogenic claudication.* Cochrane Database of Systematic Reviews, 2013(8): p. 62.

For peer review only - http://bmjopen.bmj.com/site/about/guidelines.xhtml

23. Balakatounis, K.C.P., Kalomoira A; Mitsiokapa, Evanthia A; Mavrogenis, Angoules, Antonios G; Papthanasiou, Jannis; Papgelopoulos, Panayiotis J, *Evidence-Based Evaluation and Current Practice of Non-Operative Treatment Strategies for Lumbar Stenosis*

Folia Medica, 2011. **53**(3): p. 5-14.

24. Comer, C.M., et al., *Assessment and management of neurogenic claudication associated with lumbar spinal stenosis in a UK primary care musculoskeletal service: a survey of current practice among physiotherapists.* BMC musculoskeletal disorders, 2009. **10**(1): p. 121.
25. Mansell, G., et al., *How can we design low back pain intervention studies to better explain the effects of treatment?* 2014, LWW.
26. Williamson, E., et al., *Better Outcomes for Older people with Spinal Trouble (BOOST) Trial: a randomised controlled trial of a combined physical and psychological intervention for older adults with neurogenic claudication, a protocol.* 2018. **8**(10): p. e022205.
27. Ward, L., et al., *Development and delivery of the BOOST (Better Outcomes for Older adults with Spinal Trouble) intervention for older adults with neurogenic claudication.* 2019.
28. McKillop, A.B., L.J. Carroll, and M.C. Battié, *Depression as a prognostic factor of lumbar spinal stenosis: a systematic review.* The Spine Journal, 2014. **14**(5): p. 837-846.
29. Lamb, S., et al., *A multicentred randomised controlled trial of a primary care-based cognitive behavioural programme for low back pain. The Back Skills Training (BeST) trial.* Health Technol Assess, 2010. **14**(41): p. 1-253.
30. Makris, U.E., et al., *Ageism, negative attitudes, and competing co-morbidities–why older adults may not seek care for restricting back pain: a qualitative study.* BMC geriatrics, 2015. **15**(1): p. 39.
31. Thielke, S., J. Sale, and M.C. Reid, *Aging: are these 4 pain myths complicating care?* The Journal of family practice, 2012. **61**(11): p. 666.
32. Wood, D.W., A.J. Haig, and K.S. Yamakawa, *Fear of movement/(re) injury and activity avoidance in persons with neurogenic versus vascular claudication.* The Spine Journal, 2012. **12**(4): p. 292-300.
33. Fairbank, J.C.T. and P.B. Pynsent, *The Oswestry Disability Index.* Spine, 2000. **25**(22): p. 2940-2952.
34. Danks, K.A., et al., *Relationship Between Walking Capacity, Biopsychosocial Factors, Self-efficacy, and Walking Activity in Persons Poststroke.* J Neurol Phys Ther, 2016. **40**(4): p. 232-8.
35. Harada, N.D., V. Chiu, and A.L. Stewart, *Mobility-related function in older adults: assessment with a 6-minute walk test.* Arch Phys Med Rehabil, 1999. **80**(7): p. 837-41.
36. Guralnik, J.M., et al., *Lower-extremity function in persons over the age of 70 years as a predictor of subsequent disability.* N Engl J Med, 1995. **332**(9): p. 556-61.
37. Shuval, K., et al., *Sedentary behaviour and physical inactivity assessment in primary care: the Rapid Assessment Disuse Index (RADI) study.* 2014. **48**(3): p. 250-255.
38. Stucki, G., et al., *Measurement properties of a self-administered outcome measure in lumbar spinal stenosis.* Spine, 1996. **21**(7): p. 796-803.
39. Newell, A.M., et al., *The modified gait efficacy scale: establishing the psychometric properties in older adults.* 2012. **92**(2): p. 318-328.
40. Waddell, G., et al., *A Fear-Avoidance Beliefs Questionnaire (FABQ) and the role of fear-avoidance beliefs in chronic low back pain and disability.* Pain, 1993. **52**(2): p. 157-68.
41. Conway, J., C.C. Tomkins, and A.J. Haig, *Walking assessment in people with lumbar spinal stenosis: capacity, performance, and self-report measures.* The Spine Journal, 2011. **11**(9): p. 816-823.
42. Amundsen, T., et al., *Lumbar spinal stenosis. Clinical and radiologic features.* 1995. **20**(10): p. 1178-1186.

For peer review only - http://bmjopen.bmj.com/site/about/guidelines.xhtml

43. Yamashita, K., et al., *Correlation of patient satisfaction with symptom severity and walking ability after surgical treatment for degenerative lumbar spinal stenosis.* Spine, 2003. **28**(21): p. 2477-2481.
44. Markman, J.D., et al., *Evaluation of outcome measures for neurogenic claudication: A patient-centered approach.* Neurology, 2015. **85**(14): p. 1250-6.
45. Rantakokko, M., et al., *Mobility decline in old age.* 2013. **41**(1): p. 19-25.
46. Keysor, J.J., *Does late-life physical activity or exercise prevent or minimize disablement? A critical review of the scientific evidence.* Am J Prev Med, 2003. **25**(3 Suppl 2): p. 129-36.
47. Andrews, J.S., et al., *Muscle strength, muscle mass, and physical disability in women with systemic lupus erythematosus.* Arthritis Care Res (Hoboken), 2015. **67**(1): p. 120-7.
48. Clemson, L., et al., *Integration of balance and strength training into daily life activity to reduce rate of falls in older people (the LiFE study): randomised parallel trial.* BMJ, 2012. **345**: p. e4547.
49. Hayden, J., et al., *Exercise therapy for treatment of non-specific low back pain.* Cochrane Database of Systematic Reviews, 2005(3).
50. Daniels, R., et al., *Interventions to prevent disability in frail community-dwelling elderly: a systematic review.* BMC Health Serv Res, 2008. **8**: p. 278.
51. Rantanen, T., et al., *Disability, physical activity, and muscle strength in older women: the Women's Health and Aging Study.* Archives of physical medicine and rehabilitation, 1999. **80**(2): p. 130-135.
52. Van Heuvelen, M.J., et al., *Physical fitness related to disability in older persons.* Gerontology, 2000. **46**(6): p. 333-341.
53. REJC, E., A. DEL TORTO, and S.J.A.K. LAZZER, *BENEFITS OF AEROBIC EXERCISE TRAINING WITH RECOMMENDATIONS FOR HEALTHY AGING.* 2017. **8**(2).
54. Lee, P.G., E.A. Jackson, and C.R.J.A.f.p. Richardson, *Exercise Prescriptions in Older Adults.* 2017. **95**(7).
55. Richmond, H., et al., *The Effectiveness of Cognitive Behavioural Treatment for Non-Specific Low Back Pain: A Systematic Review and Meta-Analysis.* PLoS One, 2015. **10**(8): p. e0134192.
56. Eccleston, C., A.C. Williams, and S. Morley, *Psychological therapies for the management of chronic pain (excluding headache) in adults.* Cochrane Database Syst Rev, 2009(2): p. CD007407.
57. Hoffman, B.M., et al., *Meta-analysis of psychological interventions for chronic low back pain.* Health Psychol, 2007. **26**(1): p. 1-9.
58. Wertli, M.M., et al., *Fear-avoidance beliefs—a moderator of treatment efficacy in patients with low back pain: a systematic review.* The Spine Journal, 2014. **14**(11): p. 2658-2678.
59. Mansell, G., et al., *Mediators of Treatment Effect in the Back In Action Trial: Using Latent Growth Modeling to Take Change Over Time Into Account.* Clin J Pain, 2017. **33**(9): p. 811-819.
60. Collins, T.C., S. Lunos, and J.S. Ahluwalia, *Self-efficacy is associated with walking ability in persons with diabetes mellitus and peripheral arterial disease.* Vasc Med, 2010. **15**(3): p. 189-95.
61. Karasawa, Y., et al., *Association between change in self-efficacy and reduction in disability among patients with chronic pain.* 2019. **14**(4): p. e0215404.
62. Focht, B.C., et al., *Exercise, self-efficacy, and mobility performance in overweight and obese older adults with knee osteoarthritis.* 2005. **53**(5): p. 659-665.
63. Seymour, R.B., et al., *Comparison of two methods of conducting the Fit and Strong! program.* 2009. **61**(7): p. 876-884.
64. Ammendolia, C., et al., *Nonoperative treatment for lumbar spinal stenosis with neurogenic claudication.* Cochrane Database Syst Rev, 2013(8): p. CD010712.

For peer review only - http://bmjopen.bmj.com/site/about/guidelines.xhtml

65. Whitman, J.M., et al., *A comparison between two physical therapy treatment programs for patients with lumbar spinal stenosis: a randomized clinical trial.* Spine (Phila Pa 1976), 2006. **31**(22): p. 2541-9.
66. Vincent, H.K., et al., *Resistance exercise, disability, and pain catastrophizing in obese adults with back pain.* Medicine and science in sports and exercise, 2014. **46**(9): p. 1693.
67. Booth, J., et al., *Exercise for chronic musculoskeletal pain: A biopsychosocial approach.* Musculoskeletal Care, 2017. **15**(4): p. 413-421.
68. Smith, B.E., et al., *Musculoskeletal pain and exercise-challenging existing paradigms and introducing new.* Br J Sports Med, 2018.
69. Runhaar, J., et al., *Identifying potential working mechanisms behind the positive effects of exercise therapy on pain and function in osteoarthritis; a systematic review.* Osteoarthritis Cartilage, 2015. **23**(7): p. 1071-82.
70. Tomkins, C.C., et al., *Physical therapy treatment options for lumbar spinal stenosis.* J Back Musculoskelet Rehabil, 2010. **23**(1): p. 31-7.
71. Imai, K., et al., *Unpacking the black box of causality: Learning about causal mechanisms from experimental and observational studies.* 2011. **105**(4): p. 765-789.
72. Imai, K., L. Keele, and D.J.P.m. Tingley, *A general approach to causal mediation analysis.* 2010. **15**(4): p. 309.
73. Tingley, D., et al., *Mediation: R package for causal mediation analysis.* 2014.
74. Imai, K., L. Keele, and T.J.S.s. Yamamoto, *Identification, inference and sensitivity analysis for causal mediation effects.* 2010: p. 51-71.
75. VanderWeele, T., *Explanation in causal inference: methods for mediation and interaction.* 2015: Oxford University Press.
76. Imai, K. and T.J.P.A. Yamamoto, *Identification and sensitivity analysis for multiple causal mechanisms: Revisiting evidence from framing experiments.* 2013. **21**(2): p. 141-171.
77. Buuren, S.v. and K.J.J.o.s.s. Groothuis-Oudshoorn, *mice: Multivariate imputation by chained equations in R.* 2010: p. 1-68.
78. Cleland, J.A., et al., *Psychometric properties of selected tests in patients with lumbar spinal stenosis.* 2012. **12**(10): p. 921-931.

For peer review only

For peer review only - http://bmjopen.bmj.com/site/about/guidelines.xhtml

For peer review only

For peer review only - http://bmjopen.bmj.com/site/about/guidelines.xhtml

Figure 1 Mediation protocol decision process

Figure 2 Directed acyclic graphics (DAG)

A

B

Legend for Figure 2

Blue lines represent indirect effects (mechanisms) of interest. Green lines represent direct effects (direct effect of treatment on outcome plus all unspecified indirect effects). Red lines represent possible effects that could induce confounding for indirect and direct effects. (A) A single mediator model where the intervention (X) exerts its effect on the outcome(s) Y, via an indirect path through the primary mediator (M1). (B) A serial multiple mediator model where the intervention (X) exerts its effect on the outcome (Y) via an indirect path through one of five alternative mediators (M2) and the primary mediator (M1), and a direct path (X to Y). ODI = Oswestry Disability Index, SPPB = S physical battery, RADI Rapid Assessment Disuse index, SSS = Swiss Spinal Stenosis Symptom severity scale, FABQ = fear avoidance beliefs questionnaire. M1 = 12 month measure, M2 = 6 month measures, Y = 12 month measure.

For peer review only - http://bmjopen.bmj.com/site/about/guidelines.xhtml

Table 1. Overview of Mediator Models

Model	Treatment	Alternative mediators at 6 months (M2)	Primary mediator at 6 months (M1)	Outcome at 12 months
If the total effect of the intervention on Disability outcome (ODI) is significant				
1.0	BOOST		Walking Capacity	Disability (ODI)
If the indirect effect through walking capacity is significant from model 1.0				
1.1	BOOST	Physical Function (SPPB)	Walking Capacity	Disability (ODI)
1.2	BOOST	Physical Activity (RADI)	Walking Capacity	Disability (ODI)
1.3	BOOST	Fear avoidance (FABQ)	Walking Capacity	Disability (ODI)
1.4	BOOST	Self Efficacy Walking (SE-W)	Walking Capacity	Disability (ODI)
1.5	BOOST	Symptom Severity (SSS)	Walking Capacity	Disability (ODI)
If the indirect effect through walking capacity is not significant from model 1.0				
1.6	BOOST	Physical Function (SPPB)		Disability (ODI)
1.7	BOOST	Physical Activity (RADI)		Disability (ODI)
1.8	BOOST	Fear avoidance (FABQ)		Disability (ODI)
1.9	BOOST	Self Efficacy Walking (SE-W)		Disability (ODI)
1.10	BOOST	Symptom Severity (SSS)		
If the total effect of the intervention on Disability outcome (ODI) is not significant				
2.0	BOOST		Walking Capacity	Disability (ODI)
2.1	BOOST	Physical Function (SPPB)		Disability (ODI)
2.2	BOOST	Physical Activity (RADI)		Disability (ODI)
2.3	BOOST	Fear avoidance (FABQ)		Disability (ODI)
2.4	BOOST	Self Efficacy Walking (SE-W)		Disability (ODI)
2.5	BOOST	Symptom Severity (SSS)		Disability (ODI)

Multiple mediator models will only be tested if there is a significant relationship between M1 and M2. If the relationship is non-significant, then the alternative mediators will be tested in separate single mediator models. Significance levels are set a priori at p<0.05.
ODI = Oswestry Disability Index

For peer review only - http://bmjopen.bmj.com/site/about/guidelines.xhtml

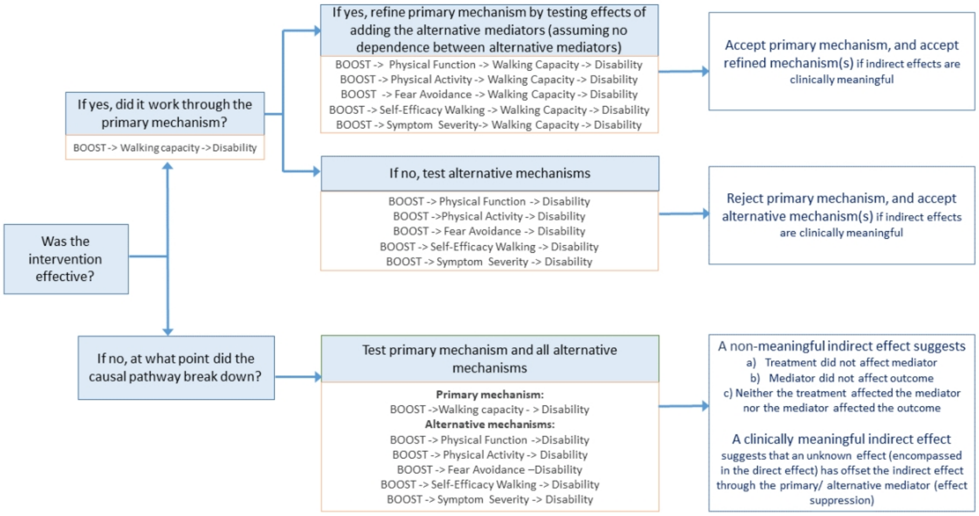

Heading for Figure 1:
Figure 1 Mediation protocol decision process

Figure 2A

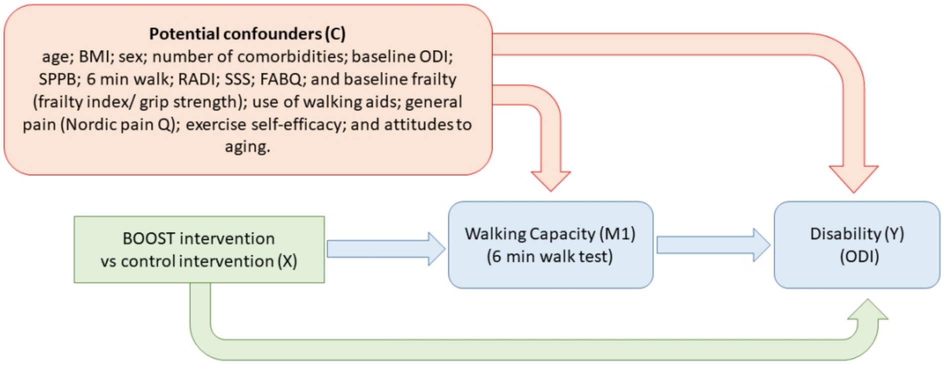

Caption: Heading below applies to Figures 2A and 2B
Figure 2 Directed acyclic graphics (DAG)

Figure 2B

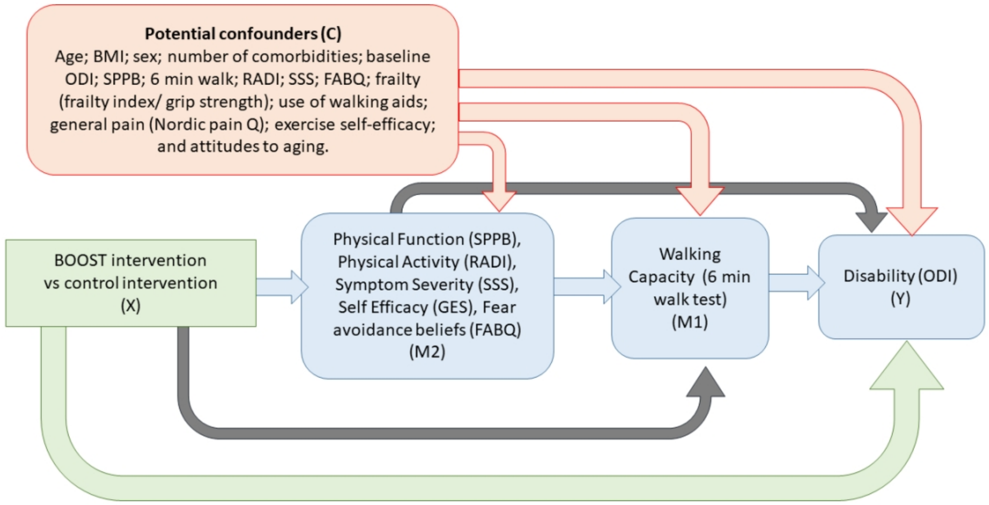

Legend below applies to Figures 2A and 2B

Blue lines represent indirect effects (mechanisms) of interest. Green lines represent direct effects (direct effect of treatment on outcome plus all unspecified indirect effects). Red lines represent possible effects that could induce confounding for indirect and direct effects. (A) A single mediator model where the intervention (X) exerts its effect on the outcome(s) Y, via an indirect path through the primary mediator (M1). (B) A serial multiple mediator model where the intervention (X) exerts its effect on the outcome (Y) via an indirect path through one of five alternative mediators (M2) and the primary mediator (M1), and a direct path (X to Y). ODI = Oswestry Disability Index, SPPB = S physical battery, RADI Rapid Assessment Disuse index, SSS = Swiss Spinal Stenosis Symptom severity scale, FABQ = fear avoidance beliefs questionnaire. M1 = 12 month measure, M2 = 6 month measures, Y = 12 month measure.

BMJ Open

Understanding the mechanisms of a combined physical and psychological intervention for people with neurogenic claudication: Protocol for a causal mediation analysis of the BOOST Trial

Journal:	*BMJ Open*
Manuscript ID	bmjopen-2020-037121.R1
Article Type:	Protocol
Date Submitted by the Author:	01-Apr-2020
Complete List of Authors:	Comer, Christine; Leeds Community Healthcare NHS Trust, Musculoskeletal and Rehabilitation Services; University of Leeds Faculty of Medicine and Health, Leeds Institute of Rheumatic and Musculoskeletal Medicine<br>Lee, Hopin; Hunter New England Population Health; University of Oxford Nuffield Department of Orthopaedics Rheumatology and Musculoskeletal Sciences<br>Williamson, Esther; Nuffield Department of Orthopaedics, Rheumatology and Musculoskeletal Sciences, University of Oxford, United Kingdom. , Centre for Rehabilitation Research in Oxford<br>Lamb, Sarah; University of Exeter, College of Medicine and Health; University of Oxford, NDORMS
**Primary Subject Heading**:	Health services research
Secondary Subject Heading:	Rehabilitation medicine
Keywords:	Spine < ORTHOPAEDIC & TRAUMA SURGERY, GERIATRIC MEDICINE, REHABILITATION MEDICINE

SCHOLARONE™
Manuscripts

I, the Submitting Author has the right to grant and does grant on behalf of all authors of the Work (as defined in the below author licence), an exclusive licence and/or a non-exclusive licence for contributions from authors who are: i) UK Crown employees; ii) where BMJ has agreed a CC-BY licence shall apply, and/or iii) in accordance with the terms applicable for US Federal Government officers or employees acting as part of their official duties; on a worldwide, perpetual, irrevocable, royalty-free basis to BMJ Publishing Group Ltd ("BMJ") its licensees and where the relevant Journal is co-owned by BMJ to the co-owners of the Journal, to publish the Work in this journal and any other BMJ products and to exploit all rights, as set out in our licence.

The Submitting Author accepts and understands that any supply made under these terms is made by BMJ to the Submitting Author unless you are acting as an employee on behalf of your employer or a postgraduate student of an affiliated institution which is paying any applicable article publishing charge ("APC") for Open Access articles. Where the Submitting Author wishes to make the Work available on an Open Access basis (and intends to pay the relevant APC), the terms of reuse of such Open Access shall be governed by a Creative Commons licence – details of these licences and which Creative Commons licence will apply to this Work are set out in our licence referred to above.

Other than as permitted in any relevant BMJ Author's Self Archiving Policies, I confirm this Work has not been accepted for publication elsewhere, is not being considered for publication elsewhere and does not duplicate material already published. I confirm all authors consent to publication of this Work and authorise the granting of this licence.

For peer review only

Understanding the mechanisms of a combined physical and psychological intervention for people with neurogenic claudication: Protocol for a causal mediation analysis of the BOOST Trial

Christine Comer [1,2], Hopin Lee [3,4] Esther Williamson [3] and Sarah Lamb [3,5]

1 Leeds Community Healthcare Musculoskeletal and Rehabilitation Services, Leeds Community Healthcare NHS Trust, Stockdale House, Victoria Road, Leeds UK

2 Leeds Institute of Rheumatic and Musculoskeletal Medicine, University of Leeds, Leeds, UK

3 Centre for Rehabilitation Research, Nuffield Department of Orthopaedics, Rheumatology and Musculoskeletal Sciences, University of Oxford, Oxford, UK

4 School of Medicine and Public Health, University of Newcastle, New South Wales, Australia

5 University of Exeter Medical School, Institute of Health Research, Devon, UK

Correspondence to Christine Comer, c.comer@leeds.ac.uk, +44 (0)113 3921521

Authors' contributions: all authors were involved in the design and write-up of this protocol.

Funding statement: Christine Comer is funded by a National Institute for Health Research (NIHR) Clinical Lectureship ICA-CL-2017-03-015 for this research project. This publication presents independent research. The views expressed are those of the author(s) and not necessarily those of the NHS, the NIHR or the Department of Health and Social Care.

Competing interests statement: None of the authors have competing interests to declare

Word count 3,131

Keywords

Mediation, Causality, Spinal Stenosis, Behaviour and behaviour mechanisms, Exercise, Rehabilitation

For peer review only - http://bmjopen.bmj.com/site/about/guidelines.xhtml

ABSTRACT

Introduction
Conservative treatments such as exercise are recommended for the management of people with neurogenic claudication from spinal stenosis. However, the effectiveness and mechanisms of effect are unknown. This protocol outlines an a-priori plan for a secondary analysis of a multi-centre randomised controlled trial of a physiotherapist-delivered, combined physical and psychological intervention (Better Outcomes for Older people with Spinal Trouble (BOOST) programme).

Methods and analyses
We will use causal mediation analysis to estimate the mechanistic effects of the BOOST programme on the primary outcome of disability (measured by the Oswestry Disability Index). The primary mechanism of interest is walking capacity, and secondary mediators include fear avoidance behaviour, walking self-efficacy, physical function, physical activity and/or symptom severity. All mediators will be measured at 6 months and the outcome will be measured at 12 months from randomisation. Patient characteristics and possible confounders of the mediator-outcome effect will be measured at baseline. Sensitivity analyses will be conducted to evaluate the robustness of the estimated effects to varying levels of residual confounding.

Ethics and Dissemination
Ethical approval was given on the 03 March 2016 (National Research Ethics Committee number: 16/LO/0349). The results of this analysis will be disseminated in peer-reviewed journals and at relevant scientific conferences.

Trial Registration number ISRCTN12698674

Strengths and Limitations of the Study
- Causal mediation analysis of clinical trials can explain how an effective intervention works, or why an ineffective intervention does not work.
- We propose exploratory analysis of several potential causal pathways based on theoretical mechanisms of action and we acknowledge the risks associated with this multiple testing.
- The overall objective of this study is to identify the mechanisms that underlie the effect of the BOOST programme.
- Understanding these treatment mechanisms will provide valuable information to guide future optimisation or implementation.

For peer review only - http://bmjopen.bmj.com/site/about/guidelines.xhtml

INTRODUCTION

Neurogenic claudication (NC) is a leading cause of disability and lost independence in older adults [1-3]. NC is the term used to describe the typical symptoms associated with lumbar spinal stenosis (LSS) [4]. These symptoms are provoked by walking, and include pain, aching, paraesthesia and fatigue which radiate from the spine into the buttocks and legs [5]. NC often leads to reduced mobility [6] contributing to disability, frailty and falls [7-12], increased risks of comorbidity, social isolation and loss of independence [13, 14]. The symptoms are thought to arise because of reduced space and subsequent compromise of the neural and vascular elements in the lumbar spine [4] resulting from degenerative changes in the aging spine [15].

LSS has become the most common reason for spinal surgery in people over 65 years of age [16]. Because of the risks, costs and unclear benefits associated with surgical treatment [16-18], it is widely accepted that non-surgical treatment should be first line care [19, 20]. Advice, exercise and self-management strategies are often used in clinical practice [21-23] but there is limited robust evidence to demonstrate their efficacy and effectiveness [17].

The BOOST trial is a large multicentre RCT testing the effectiveness of a 12-week combined physical and psychological intervention delivered by physiotherapists in a group setting. The primary focus of this randomised trial is to estimate the effect of the BOOST intervention on patient-reported disability. There is, however, growing recognition of the importance of evaluating hypothesised treatment mechanisms in randomised trials [24]. Doing so can explain how an effective intervention works, or why an ineffective intervention does not work.

OBJECTIVES

We present an a priori protocol for a secondary analysis of the BOOST trial to evaluate the effects on the primary mediator of a change in walking capacity, and to explore alternative mediators of changes in fear avoidance behaviour, walking self-efficacy, physical function, physical activity, and symptom severity. The aim of this planned analysis is to contribute to our understanding of treatment mechanisms and provide valuable information to guide future intervention development and implementation.

METHODS AND ANALYSES

Design

This study is a causal mediation analysis of the National Institute of Health Research (NIHR) funded BOOST trial. The BOOST trial [ISRCTN12698674] is a high-quality multi-centred randomised controlled trial. It aims to evaluate the clinical- and cost-effectiveness of a physiotherapist-delivered, combined physical and psychological intervention for community-dwelling older adults with symptoms of NC, compared to

For peer review only - http://bmjopen.bmj.com/site/about/guidelines.xhtml

a control treatment of best practice (standardised advice and education). BOOST trial details are published separately in the trial protocol [25].

Patient and public involvement

Patient and public involvement has been embedded in the BOOST trial and is described fully in the trial protocol [25]

Participants

438 participants with symptoms of NC from across 15 primary- and secondary- care centres in the UK have been randomised to the trial intervention or control treatment at a 2:1 ratio. We recruited participants over the age of 65 years from spinal clinics within primary and secondary care centres (n=394) and via a primary care survey of community dwelling older adults (n=44). They were eligible for the study if they presented with symptoms consistent with NC (back and/or leg pain or symptoms aggravated by standing or walking and eased by sitting or bending) and were able to participate safely in the group rehabilitation programme.

BOOST trial Intervention

A detailed description of the BOOST intervention and its development have been published elsewhere [26]. In summary, the BOOST intervention combines physical and psychological components in a programme delivered to small groups of participants over twelve sessions. The physical component comprises a 60-minute exercise programme at each session, including exercises for strength, balance and flexibility, and a walking circuit. The level of exercise difficulty is individually tailored, and is progressed over the 12-week programme by increasing the number of repetitions, sets, and increasing weights and/ or speed. The psychological component consists of a series of 30-minute discussions at each of the twelve sessions. Early sessions are aimed at facilitating self-efficacy and safety, including addressing unhelpful pain behaviour and fear-related avoidance of movement; providing simple concepts of exercise physiology relevant to improving mobility; and introducing basic behavioural techniques of pacing and graded activity. Subsequent sessions use peer-support to encourage exercise adherence, identify facilitators and barriers, and reinforce long-term exercise adherence through peer discussion about exercise confidence and motivation, access to local activities, and advice on coping with acute symptom flare-ups. Over the 12-week supervised programme, session frequency reduces from twice weekly to once weekly and then to once fortnightly. Concurrently, home exercise programmes are introduced with the aim of encouraging participants to increase their level of independent, unsupervised exercise and physical activity at home.

We developed the intervention collaboratively with clinical experts and patients to address some of the unique challenges of managing back problems in older people. These include age-related physical changes of reduced muscle strength and fitness, in addition to psychological factors such as depression [27] and unhelpful behaviours and beliefs about aging and exercise which are known to negatively impact on outcomes and act as barriers to accessing and engaging with treatment [28-31].

Control intervention

For peer review only - http://bmjopen.bmj.com/site/about/guidelines.xhtml

The control intervention consists of a one-hour individual session with a physiotherapist for assessment, advice and education about NC and being physically active, advice on exercises including flexion-based exercises, use of medications and when to seek more advice. The advice and education are reinforced in a leaflet and a further two review appointments are available if required.

Outcomes

The Oswestry Disability Index (ODI) [32] is the primary outcome measure for the BOOST Trial. The ODI (also known as the Oswestry Low Back Pain Disability Questionnaire) is the most widely used measure in low back pain research and is considered the 'gold standard' of low back functional outcome tools. It is commonly used to provide a patient reported measure of walking limitation and other functional limitations in Lumbar spinal stenosis trials, alongside objective measures of walking ability [33].

The ODI comprises a series of questions about how pain impacts on the patient's ability to carry out a range of daily functions. For each question, possible answers are scored on a scale of 0 to 5. These scores are summed and expressed as a percentage of the maximum possible score for disability, with higher scores representing worse disability. In the BOOST trial, ODI scores are measured for each participant at baseline, and at 6 and 12 months after randomisation.

Important secondary outcome measures for the BOOST trial focus on walking capacity, and on fear avoidance behaviour, walking self-efficacy, physical function, physical activity and symptom severity: The six-minute walk test provides an objective measure of walking capacity and entails a timed measure of walking distance which is easily performed in the clinical setting [34]. The Short Physical Performance Battery (SPPB) provides an objective measure of physical performance which incorporates a balance test, a functional lower limb strength test of 5 timed chair stands, and a timed walking test over 8 feet. This is similarly easy to complete in a clinic [35]. Outcome questionnaires completed by participants provide subjective evaluation of relevant constructs. These include physical activity measured by a modified version of the Rapid Assessment Disuse Index (RADI) [36]; severity of stenotic symptoms measured by the Swiss Spinal Stenosis Questionnaire (SSS) symptom severity subscale [37]; walking self-efficacy measured by a single item from the Modified Gait Efficacy Scale (GES) [38]; and fear of movement measured by the physical activity subscale of the Fear Avoidance Beliefs Questionnaire (FABQ) [39]. These outcomes are all measured at baseline, and at 6 and 12 months following randomisation.

Causal Mediation Analysis - Rationale

We will test the mechanisms of the BOOST intervention for patients with NC by estimating the extent to which different potential mediators explain the effect of the BOOST intervention on participants' ODI outcome scores. This will be achieved by deconstructing the total effect of the BOOST treatment into indirect and direct effects. The indirect effect is the path through which the intervention affects the outcome via a selected mediator. The direct effect is the remaining total effect that is not encompassed by the indirect effect.

For peer review only - http://bmjopen.bmj.com/site/about/guidelines.xhtml

Walking capacity has been selected as the primary mediator for our causal mediation analysis model. Walking limitation is a hallmark of disability in LSS [40] and a serious concern for people with LSS who often seek healthcare because of reduced mobility [41-43]. The BOOST intervention incorporates both physical and cognitive behavioural components aimed at reducing disability, and increased walking is a key target.

The BOOST intervention is underpinned by a strong theoretical premise for addressing age-related decline in mobility. Exercise components of the intervention aim to improve physical function by targeting reduced muscle strength, balance and sensory impairments [44-53]. Whilst cognitive behavioural approaches have not been well investigated in people with LSS [54], this is an important component of the BOOST intervention based on evidence that addressing unhelpful beliefs and behaviours can mediate changes in both disability and symptom severity in low back pain populations [55-60]. In addition, self-efficacy is likely to play a role in mediating disability, physical function and activity in older adults with chronic musculoskeletal conditions [61-63]. Alternative mediators that we will include in our causal mediation models therefore include physical function (SPPB), physical activity (RADI), fear avoidance behaviour (FABQ), and walking self-efficacy (GES).

Our final alternative mediator is symptom severity (SSS). Little is known about how exercise affects NC symptom severity [64]. In theory, better muscular strength, control and endurance, and increased flexibility are likely not only to enhance physical ability [65, 66], but also to reduce pain by altering the loading of the musculoskeletal system and changing central pain processes and immune system responses [67-69]. Neuroischaemic lower limb symptoms such as paraesthesia, numbness, sensory and balance changes might also be improved through reduced nerve root ischaemia and venous pooling as a result of exercise [4, 70].

To understand the mechanisms of the BOOST intervention, we plan to test our primary hypothesis that changes in disability will be mediated primarily by changes in walking capacity, and that various mechanisms might explain these changes in walking capacity: We hypothesise that the progressive exercise component of the BOOST intervention will exert its effect on walking capacity via initial changes in physical function and physical activity levels during treatment. We further hypothesise that the cognitive behavioural components of the intervention will exert effects on walking capacity through reductions in fear avoidance behaviour and increased self-efficacy. There is also the potential for both the exercise and the cognitive behavioural components of the intervention to reduce symptom severity, resulting in increased walking capacity.

Our secondary hypothesis is that the causal effects of the BOOST intervention on disability will be explained through changes in fear avoidance behaviour, self-efficacy, physical function, physical activity and/or symptom severity (SSS) without exerting any effects on walking capacity.

If the BOOST intervention is found to be effective, causal mediation analysis will explain how the intervention works. Conversely, if the intervention is not found to be

For peer review only - http://bmjopen.bmj.com/site/about/guidelines.xhtml

effective, causal mediation analysis will identify where the hypothesised mechanism broke down [71].

Causal Mediation analysis - models

The decision-making process that will guide our analyses is laid out in Figure 1. Table 1 provides an overview of the planned mediation models contingent on whether or not the BOOST intervention is effective:

► If the intervention is effective, our primary objective is to estimate the extent to which the joint effects of walking capacity, fear avoidance behaviour, walking self-efficacy, physical function, physical activity, and symptom severity mediate this effect.

Our secondary objective will be to further refine this model through the candidate primary mediator, walking capacity; and via five secondary mediators (fear avoidance behaviour, walking self-efficacy, physical function, physical activity, and symptom severity). If the indirect effect through the primary mediator is significant, we will consider sequentially ordered multiple mediator models through secondary mediators. The serial multiple mediator models accounting for potential confounders are presented in the Directed Acyclic Graphics (DAGs) in Figure 2.

► If the intervention is not effective, our primary objective is to determine where the causal paths break down. All potential mediators (walking capacity, fear avoidance, self-efficacy, physical function, physical activity and symptom severity) will be evaluated.

If alternative pathways of mediation emerge during the analysis of the trial and associated qualitative data these will also be investigated. The publication of this protocol does not preclude other analyses if indicated.

Statistical Analysis

We will use a model-based inference approach for causal mediation analysis [72]. All analyses will be conducted in R (The R Foundation for Statistical Computing) using the "*medflex*" and "*mediation*" packages [73, 74].

To estimate a joint indirect effect, we will consider a natural indirect effect of the intervention on the outcome that is exerted through a vector of all six mediators (walking capacity, fear avoidance behaviour, walking self-efficacy, physical function, physical activity, and symptom severity). The advantage of this approach is that we can consider all possible mechanisms simultaneously and relax the assumption of omitting confounders of the mediator-outcome effect that is affected by randomisation. We will use the imputation-based approach outlined by Steen et al [75, 76] to fit a natural effect model with robust standard errors based on the sandwich-estimator – the recommended approach when the ordering of multiple mediators is unknown.

For peer review only - http://bmjopen.bmj.com/site/about/guidelines.xhtml

To consider the mediators independently, we will construct single mediator models for the ODI outcome. For each single mediator model (primary mediator model of walking capacity, and secondary mediator models of fear avoidance, self-efficacy, physical function, physical activity and symptom severity), we will estimate the intervention-mediator effect, the mediator-outcome effect, the average causal mediation effect (ACME), average direct effect (ADE) and the average total effect (ATE). We will also estimate the proportion mediated in each single mediator model. The ACME is the average intervention effect through the mediator; ADE is the average intervention effect that works through all other mechanism excluding the selected mediator; and the ATE is the average effect of the intervention on the outcome. The ATE is the sum of the ACME and ADE on the additive scale. The proportion mediated is the fraction of the ATE that is explained by the ACME.

We will fit two regression models: the mediator model, and the outcome model. The mediator model will use linear regression with treatment allocation as the independent variable, and the mediator as the dependent variable, with the baseline level of the mediator as a covariate. The outcome model for disability will also use linear regression. Each outcome model will be constructed with the mediator as the independent variable, the outcome as the dependent variable, and treatment allocation, baseline values of the meditator and outcome variable in addition to the set of observed pre-treatment confounders as covariates. Because the mediators are not randomised, it is possible for the mediator-outcome effects to be confounded. To address this problem, we will adjust for the effects of pre-treatment confounders based on theorised causal relationships with the mediators and outcome variables from expert clinical knowledge and available evidence. These potential confounders include age, BMI, sex, number of comorbidities, in addition to baseline measures of disability, physical function, walking capacity, physical activity, symptom severity and fear-avoidance behaviour, and baseline frailty (frailty index/ grip strength); use of walking aids; general pain (Nordic pain Q); exercise self-efficacy; and attitudes to aging. We will also conduct a sensitivity analysis to determine the robustness of the estimated ACME and ADE to possible unmeasured confounding [77]. We will also include an interaction term (treatment allocation X mediator) in the outcome models to allow for an intervention-mediator interaction effect on the outcome and to increase model flexibility [78].

Using mediator and outcome regression models, we will simulate potential values of the mediator for each participant under each level of the intervention; then simulate potential outcome values for each participant under all combinations of the intervention and simulated mediator values. From these observed and simulated potential values of the mediator and outcome, we will calculate point estimates for the ACME, ADE, and TE and their 95% confidence intervals using 1000 bootstrapped simulations with the percentile method.

For multiple mediator models, we will use an expanded framework. [79] Multiple mediator models will only be constructed if the alternative mediator (fear avoidance, self-efficacy, physical function, physical activity and symptom severity) and primary mediator (walking capacity) are associated [79]. We will use the *multimed* function from the *mediation* package to estimate the ACME and ADE, and the sensitivity parameters.

For peer review only - http://bmjopen.bmj.com/site/about/guidelines.xhtml

We will assess the proportion of missing mediator and outcome data. We will conduct all analyses on complete cases if the proportion of missing data is <5%. If missing data exceeds 5% we will use multiple imputation by chain equations to impute 10 datasets using the “mice” package [80].

To facilitate clinical interpretation, we will calculate how much change in a given mediator would equate to a minimally clinically important difference (MCID) of disability (5 point change in ODI score [81]) by using the regression coefficients from the outcome model, we will divide the MCID by the mediator coefficient and 95% Confidence Interval from the outcome regression model.

DISCUSSION

We do not currently know whether non-surgical interventions are effective in reducing disability reported by older people with NC symptoms. The BOOST trial will provide important high-quality evidence about the effectiveness of an intervention incorporating components to address both physical and psychological impairments. The targets of this comprehensive intervention include walking capacity, physical function, physical activity, self-efficacy, unhelpful beliefs about activity, and symptom severity. We cannot assume, however, that if the intervention improves any of these variables the participants will experience benefits in patient-reported outcomes such as the ODI.

Mediation analysis conducted in parallel to an RCT can address specific questions about the underlying mechanisms of the intervention being tested. This protocol describes the planned causal mediation analysis that we will undertake to evaluate hypothetical mechanisms that underlie the effect of the BOOST rehabilitation programme. This will help us to understand how the BOOST intervention works, or doesn’t work.

If the BOOST intervention is found to be effective and this mediation analysis indicates that improvements in ODI scores are mediated by walking capacity, which in turn is mediated by improved self-efficacy, then this provides valuable information for refining the intervention with greater focus on self-efficacy to optimise treatment effectiveness. If, on the other hand, the BOOST trial is not found to be produce a significant improvement in patient reported disability, then the analysis will help us to understand where the hypothesises causal mechanisms of treatment break down. For example, we may find that although the BOOST intervention improves walking capacity, this does not translate into an improvement in ODI score and may therefore not be an appropriate treatment target. The causal analysis will therefore provide valuable information for designing, refining and implementing an effective intervention.

Because this is an exploratory study we have selected several mediators based on our current clinical understanding and evidence for exercise and behavioural treatments. Several potential causal pathways will therefore be explored, and we acknowledge the risks associated with this multiple testing. If alternative pathways of mediation emerge during the analysis of the trial and associated qualitative data, we will investigate these further. The publication of this protocol does not preclude other

For peer review only - http://bmjopen.bmj.com/site/about/guidelines.xhtml

analyses if indicated. Future studies may be needed to further explore the inter-relationship of the mediators explored in our analyses.

CONCLUSIONS

Clinical trials do not address questions around *how* an intervention affects outcomes, and therefore may not identify the most effective treatment targets for a particular patient population. Mediation analysis of clinical trials can estimate how much the total effect of the treatment can be attributed to pre-specified treatment targets. Improving our understanding of these treatment mechanisms can generate evidence that can be used to tailor and refine treatments and optimise clinical effectiveness. In this study, we will estimate the causal mediation effects of a rehabilitation programme incorporating exercise and behavioural components for patients with NC.

Ethics: Ethical approval was given on the 03 March 2016 (National Research Ethics Committee number: 16/LO/0349).

Acknowledgements: We thank Judith Fitch, lead PPI representative and a co-applicant who contributed to the design of the primary trial, and the patient and public involvement representatives who have provided advice and feedback on the development of the BOOST intervention, patient materials and conduct of the trial.

Authors' contributions: CC, HL, EW and SL conceived and designed the study. SL and EW led the primary trial and data acquisition. SL is the Chief Investigator. HL, EW, CC, SL contributed to the analytical plan. CC led the preparation of the draft and HL, EW, SL edited and contributed to the final manuscript.

Funding statement: Christine Comer is funded by a National Institute for Health Research (NIHR) Clinical Lectureship ICA-CL-2017-03-015 for this research project. This publication presents independent research. The views expressed are those of the author(s) and not necessarily those of the NHS, the NIHR or the Department of Health and Social Care.

Competing interests statement: None of the authors have competing interests to declare

For peer review only - http://bmjopen.bmj.com/site/about/guidelines.xhtml

REFERENCES

1. Ishimoto, Y., et al., *Prevalence of symptomatic lumbar spinal stenosis and its association with physical performance in a population-based cohort in Japan: the Wakayama Spine Study.* Osteoarthritis Cartilage, 2012. **20**(10): p. 1103-8.
2. Fanuele, J.C., et al., *The impact of spinal problems on the health status of patients: have we underestimated the effect?* Spine (Phila Pa 1976), 2000. **25**(12): p. 1509-14.
3. Winter, C.C., et al., *Walking ability during daily life in patients with osteoarthritis of the knee or the hip and lumbar spinal stenosis: a cross sectional study.* BMC Musculoskelet Disord, 2010. **11**: p. 233.
4. Lurie, J. and C. Tomkins-Lane, *Management of lumbar spinal stenosis.* BMJ, 2016. **352**: p. h6234.
5. Kreiner, D., et al., *Evidence-Based Clinical Guidelines for Multidisciplinary Spine Care.* 2011: p. 43-44.
6. Ammendolia, C., et al., *What interventions improve walking ability in neurogenic claudication with lumbar spinal stenosis? A systematic review.* 2014. **23**(6): p. 1282-1301.
7. Eggermont, L.H., R.H. Shmerling, and S.G. Leveille, *Tender point count, pain, and mobility in the older population: the mobilize Boston study.* The Journal of Pain, 2010. **11**(1): p. 62-70.
8. Koponen, M.P., et al., *Analgesic use and frailty among community-dwelling older people.* Drugs & aging, 2013. **30**(2): p. 129-136.
9. Leveille, S.G., et al., *The pathway from musculoskeletal pain to mobility difficulty in older disabled women.* Pain, 2007. **128**(1-2): p. 69-77.
10. Leveille, S.G., et al., *Chronic musculoskeletal pain and the occurrence of falls in an older population.* Jama, 2009. **302**(20): p. 2214-2221.
11. Makris, U.E., et al., *Restricting Back Pain and Subsequent Mobility Disability in Community-Living Older Persons.* Journal of the American Geriatrics Society, 2014. **62**(11): p. 2142-2147.
12. Shega, J.W., et al., *Persistent pain and frailty: a case for homeostenosis.* Journal of the American Geriatrics Society, 2012. **60**(1): p. 113-117.
13. Fried, L.P., et al., *Frailty in older adults: evidence for a phenotype.* The Journals of Gerontology Series A: Biological Sciences and Medical Sciences, 2001. **56**(3): p. M146-M157.
14. Troutman-Jordan, M. and J. Staples, *Successful aging from the viewpoint of older adults.* Res Theory Nurs Pract, 2014. **28**(1): p. 87-104.
15. Chad, D.A.J.N.c., *Lumbar spinal stenosis.* 2007. **25**(2): p. 407-418.
16. Deyo, R.A., et al., *Trends, major medical complications, and charges associated with surgery for lumbar spinal stenosis in older adults.* JAMA, 2010. **303**(13): p. 1259-65.
17. Zaina, F., et al., *Surgical Versus Nonsurgical Treatment for Lumbar Spinal Stenosis.* Spine, 2016. **41**(14): p. E857-E868.
18. Kovacs, F.M., G. Urrutia, and J.D. Alarcon, *Surgery versus conservative treatment for symptomatic lumbar spinal stenosis: a systematic review of randomized controlled trials.* Spine (Phila Pa 1976), 2011. **36**(20): p. E1335-51.
19. Ammendolia, C., et al., *Nonoperative treatment for lumbar spinal stenosis with neurogenic claudication.* Cochrane Database of Systematic Reviews, 2013(8): p. 62.
20. Rousing, R., et al., *Danish national clinical guidelines for surgical and nonsurgical treatment of patients with lumbar spinal stenosis.* Eur Spine J, 2019. **28**(6): p. 1386-1396.
21. Balakatounis, K.C.P., Kalomoira A; Mitsiokapa, Evanthia A; Mavrogenis, Angoules, Antonios G; Papthanasiou, Jannis; Papgelopoulos, Panayiotis J, *Evidence-Based Evaluation and Current Practice of Non-Operative Treatment Strategies for Lumbar Stenosis*

Folia Medica, 2011. **53**(3): p. 5-14.

For peer review only - http://bmjopen.bmj.com/site/about/guidelines.xhtml

22. Comer, C.M., et al., *Assessment and management of neurogenic claudication associated with lumbar spinal stenosis in a UK primary care musculoskeletal service: a survey of current practice among physiotherapists.* BMC musculoskeletal disorders, 2009. **10**(1): p. 121.
23. Matsuwaka, S.T., B.C.J.C.P.M. Liem, and R. Reports, *The Role of Exercise in Treatment of Lumbar Spinal Stenosis Symptoms.* 2018. **6**(1): p. 36-44.
24. Mansell, G., et al., *How can we design low back pain intervention studies to better explain the effects of treatment?* 2014, LWW.
25. Williamson, E., et al., *Better Outcomes for Older people with Spinal Trouble (BOOST) Trial: a randomised controlled trial of a combined physical and psychological intervention for older adults with neurogenic claudication, a protocol.* 2018. **8**(10): p. e022205.
26. Ward, L., et al., *Development and delivery of the BOOST (Better Outcomes for Older adults with Spinal Trouble) intervention for older adults with neurogenic claudication.* 2019.
27. McKillop, A.B., L.J. Carroll, and M.C. Battié, *Depression as a prognostic factor of lumbar spinal stenosis: a systematic review.* The Spine Journal, 2014. **14**(5): p. 837-846.
28. Lamb, S., et al., *A multicentred randomised controlled trial of a primary care-based cognitive behavioural programme for low back pain. The Back Skills Training (BeST) trial.* Health Technol Assess, 2010. **14**(41): p. 1-253.
29. Makris, U.E., et al., *Ageism, negative attitudes, and competing co-morbidities–why older adults may not seek care for restricting back pain: a qualitative study.* BMC geriatrics, 2015. **15**(1): p. 39.
30. Thielke, S., J. Sale, and M.C. Reid, *Aging: are these 4 pain myths complicating care?* The Journal of family practice, 2012. **61**(11): p. 666.
31. Wood, D.W., A.J. Haig, and K.S. Yamakawa, *Fear of movement/(re) injury and activity avoidance in persons with neurogenic versus vascular claudication.* The Spine Journal, 2012. **12**(4): p. 292-300.
32. Fairbank, J.C.T. and P.B. Pynsent, *The Oswestry Disability Index.* Spine, 2000. **25**(22): p. 2940-2952.
33. Jespersen, A.B. and M. Gustafsson, *Correlation between the Oswestry Disability Index and objective measurements of walking capacity and performance in patients with lumbar spinal stenosis: a systematic literature review.* Eur Spine J, 2018. **27**(7): p. 1604-1613.
34. Harada, N.D., V. Chiu, and A.L. Stewart, *Mobility-related function in older adults: assessment with a 6-minute walk test.* Arch Phys Med Rehabil, 1999. **80**(7): p. 837-41.
35. Guralnik, J.M., et al., *Lower-extremity function in persons over the age of 70 years as a predictor of subsequent disability.* N Engl J Med, 1995. **332**(9): p. 556-61.
36. Shuval, K., et al., *Sedentary behaviour and physical inactivity assessment in primary care: the Rapid Assessment Disuse Index (RADI) study.* 2014. **48**(3): p. 250-255.
37. Stucki, G., et al., *Measurement properties of a self-administered outcome measure in lumbar spinal stenosis.* Spine, 1996. **21**(7): p. 796-803.
38. Newell, A.M., et al., *The modified gait efficacy scale: establishing the psychometric properties in older adults.* 2012. **92**(2): p. 318-328.
39. Waddell, G., et al., *A Fear-Avoidance Beliefs Questionnaire (FABQ) and the role of fear-avoidance beliefs in chronic low back pain and disability.* Pain, 1993. **52**(2): p. 157-68.
40. Conway, J., C.C. Tomkins, and A.J. Haig, *Walking assessment in people with lumbar spinal stenosis: capacity, performance, and self-report measures.* The Spine Journal, 2011. **11**(9): p. 816-823.
41. Amundsen, T., et al., *Lumbar spinal stenosis. Clinical and radiologic features.* 1995. **20**(10): p. 1178-1186.
42. Yamashita, K., et al., *Correlation of patient satisfaction with symptom severity and walking ability after surgical treatment for degenerative lumbar spinal stenosis.* Spine, 2003. **28**(21): p. 2477-2481.

For peer review only

For peer review only - http://bmjopen.bmj.com/site/about/guidelines.xhtml

43. Markman, J.D., et al., *Evaluation of outcome measures for neurogenic claudication: A patient-centered approach.* Neurology, 2015. **85**(14): p. 1250-6.
44. Rantakokko, M., et al., *Mobility decline in old age.* 2013. **41**(1): p. 19-25.
45. Keysor, J.J., *Does late-life physical activity or exercise prevent or minimize disablement? A critical review of the scientific evidence.* Am J Prev Med, 2003. **25**(3 Suppl 2): p. 129-36.
46. Andrews, J.S., et al., *Muscle strength, muscle mass, and physical disability in women with systemic lupus erythematosus.* Arthritis Care Res (Hoboken), 2015. **67**(1): p. 120-7.
47. Clemson, L., et al., *Integration of balance and strength training into daily life activity to reduce rate of falls in older people (the LiFE study): randomised parallel trial.* BMJ, 2012. **345**: p. e4547.
48. Hayden, J., et al., *Exercise therapy for treatment of non-specific low back pain.* Cochrane Database of Systematic Reviews, 2005(3).
49. Daniels, R., et al., *Interventions to prevent disability in frail community-dwelling elderly: a systematic review.* BMC Health Serv Res, 2008. **8**: p. 278.
50. Rantanen, T., et al., *Disability, physical activity, and muscle strength in older women: the Women's Health and Aging Study.* Archives of physical medicine and rehabilitation, 1999. **80**(2): p. 130-135.
51. Van Heuvelen, M.J., et al., *Physical fitness related to disability in older persons.* Gerontology, 2000. **46**(6): p. 333-341.
52. REJC, E., A. DEL TORTO, and S.J.A.K. LAZZER, *BENEFITS OF AEROBIC EXERCISE TRAINING WITH RECOMMENDATIONS FOR HEALTHY AGING.* 2017. **8**(2).
53. Lee, P.G., E.A. Jackson, and C.R.J.A.f.p. Richardson, *Exercise Prescriptions in Older Adults.* 2017. **95**(7).
54. Richmond, H., et al., *The Effectiveness of Cognitive Behavioural Treatment for Non-Specific Low Back Pain: A Systematic Review and Meta-Analysis.* PLoS One, 2015. **10**(8): p. e0134192.
55. Eccleston, C., A.C. Williams, and S. Morley, *Psychological therapies for the management of chronic pain (excluding headache) in adults.* Cochrane Database Syst Rev, 2009(2): p. CD007407.
56. Hoffman, B.M., et al., *Meta-analysis of psychological interventions for chronic low back pain.* Health Psychol, 2007. **26**(1): p. 1-9.
57. Wertli, M.M., et al., *Fear-avoidance beliefs—a moderator of treatment efficacy in patients with low back pain: a systematic review.* The Spine Journal, 2014. **14**(11): p. 2658-2678.
58. Mansell, G., et al., *Mediators of Treatment Effect in the Back In Action Trial: Using Latent Growth Modeling to Take Change Over Time Into Account.* Clin J Pain, 2017. **33**(9): p. 811-819.
59. Collins, T.C., S. Lunos, and J.S. Ahluwalia, *Self-efficacy is associated with walking ability in persons with diabetes mellitus and peripheral arterial disease.* Vasc Med, 2010. **15**(3): p. 189-95.
60. Danks, K.A., et al., *Relationship Between Walking Capacity, Biopsychosocial Factors, Self-efficacy, and Walking Activity in Persons Poststroke.* J Neurol Phys Ther, 2016. **40**(4): p. 232-8.
61. Karasawa, Y., et al., *Association between change in self-efficacy and reduction in disability among patients with chronic pain.* 2019. **14**(4): p. e0215404.
62. Focht, B.C., et al., *Exercise, self-efficacy, and mobility performance in overweight and obese older adults with knee osteoarthritis.* 2005. **53**(5): p. 659-665.
63. Seymour, R.B., et al., *Comparison of two methods of conducting the Fit and Strong! program.* 2009. **61**(7): p. 876-884.
64. Ammendolia, C., et al., *Nonoperative treatment for lumbar spinal stenosis with neurogenic claudication.* Cochrane Database Syst Rev, 2013(8): p. CD010712.

For peer review only - http://bmjopen.bmj.com/site/about/guidelines.xhtml

65. Whitman, J.M., et al., *A comparison between two physical therapy treatment programs for patients with lumbar spinal stenosis: a randomized clinical trial.* Spine (Phila Pa 1976), 2006. **31**(22): p. 2541-9.
66. Vincent, H.K., et al., *Resistance exercise, disability, and pain catastrophizing in obese adults with back pain.* Medicine and science in sports and exercise, 2014. **46**(9): p. 1693.
67. Booth, J., et al., *Exercise for chronic musculoskeletal pain: A biopsychosocial approach.* Musculoskeletal Care, 2017. **15**(4): p. 413-421.
68. Smith, B.E., et al., *Musculoskeletal pain and exercise-challenging existing paradigms and introducing new.* Br J Sports Med, 2018.
69. Runhaar, J., et al., *Identifying potential working mechanisms behind the positive effects of exercise therapy on pain and function in osteoarthritis; a systematic review.* Osteoarthritis Cartilage, 2015. **23**(7): p. 1071-82.
70. Tomkins, C.C., et al., *Physical therapy treatment options for lumbar spinal stenosis.* J Back Musculoskelet Rehabil, 2010. **23**(1): p. 31-7.
71. Imai, K., et al., *Unpacking the black box of causality: Learning about causal mechanisms from experimental and observational studies.* 2011. **105**(4): p. 765-789.
72. Imai, K., L. Keele, and D.J.P.m. Tingley, *A general approach to causal mediation analysis.* 2010. **15**(4): p. 309.
73. Tingley, D., et al., *Mediation: R package for causal mediation analysis.* 2014.
74. Steen, J., et al., *Medflex: an R package for flexible mediation analysis using natural effect models.* 2017. **76**(11).
75. Steen, J., et al. *Medflex: flexible mediation analysis using natural effect models in R*. in *UK Causal Inference Meeting*. 2015.
76. VanderWeele, T. and S.J.E.m. Vansteelandt, *Mediation analysis with multiple mediators.* 2014. **2**(1): p. 95-115.
77. Imai, K., L. Keele, and T.J.S.s. Yamamoto, *Identification, inference and sensitivity analysis for causal mediation effects.* 2010: p. 51-71.
78. VanderWeele, T., *Explanation in causal inference: methods for mediation and interaction*. 2015: Oxford University Press.
79. Imai, K. and T.J.P.A. Yamamoto, *Identification and sensitivity analysis for multiple causal mechanisms: Revisiting evidence from framing experiments.* 2013. **21**(2): p. 141-171.
80. Buuren, S.v. and K.J.J.o.s.s. Groothuis-Oudshoorn, *mice: Multivariate imputation by chained equations in R.* 2010: p. 1-68.
81. Cleland, J.A., et al., *Psychometric properties of selected tests in patients with lumbar spinal stenosis.* 2012. **12**(10): p. 921-931.

For peer review only - http://bmjopen.bmj.com/site/about/guidelines.xhtml

Figure 1 Mediation protocol decision process

Figure 2 Directed acyclic graphics (DAG)

Legend for Figure 2

Blue lines represent indirect effects (mechanisms) of interest. Green lines represent direct effects (direct effect of treatment on outcome plus all unspecified indirect effects). Red lines represent possible effects that could induce confounding for indirect and direct effects. (A) A single mediator model where the intervention (X) exerts its effect on the outcome(s) Y, via an indirect path through the primary mediator (M1). (B) A serial multiple mediator model where the intervention (X) exerts its effect on the outcome (Y) via an indirect path through one of five alternative mediators (M2) and the primary mediator (M1), and a direct path (X to Y). ODI = Oswestry Disability Index, SPPB = S physical battery, RADI Rapid Assessment Disuse index, SSS = Swiss Spinal Stenosis Symptom severity scale, FABQ = fear avoidance beliefs questionnaire. M1 = 12 month measure, M2 = 6 month measures, Y = 12 month measure.

For peer review only - http://bmjopen.bmj.com/site/about/guidelines.xhtml

Table 1. Overview of Mediator Models

Model	Treatment	Alternative mediators at 6 months (M2)	Primary mediator at 6 months (M1)	Outcome at 12 months
If the total effect of the intervention on Disability outcome (ODI) is significant				
1.0	BOOST		Walking Capacity	Disability (ODI)
If the indirect effect through walking capacity is significant from model 1.0				
1.1	BOOST	Physical Function (SPPB)	Walking Capacity	Disability (ODI)
1.2	BOOST	Physical Activity (RADI)	Walking Capacity	Disability (ODI)
1.3	BOOST	Fear avoidance (FABQ)	Walking Capacity	Disability (ODI)
1.4	BOOST	Self Efficacy Walking (SE-W)	Walking Capacity	Disability (ODI)
1.5	BOOST	Symptom Severity (SSS)	Walking Capacity	Disability (ODI)
If the indirect effect through walking capacity is not significant from model 1.0				
1.6	BOOST	Physical Function (SPPB)		Disability (ODI)
1.7	BOOST	Physical Activity (RADI)		Disability (ODI)
1.8	BOOST	Fear avoidance (FABQ)		Disability (ODI)
1.9	BOOST	Self Efficacy Walking (SE-W)		Disability (ODI)
1.10	BOOST	Symptom Severity (SSS)		
If the total effect of the intervention on Disability outcome (ODI) is not significant				
2.0	BOOST		Walking Capacity	Disability (ODI)
2.1	BOOST	Physical Function (SPPB)		Disability (ODI)
2.2	BOOST	Physical Activity (RADI)		Disability (ODI)
2.3	BOOST	Fear avoidance (FABQ)		Disability (ODI)
2.4	BOOST	Self Efficacy Walking (SE-W)		Disability (ODI)
2.5	BOOST	Symptom Severity (SSS)		Disability (ODI)

Multiple mediator models will only be tested if there is a significant relationship between M1 and M2. If the relationship is non-significant, then the alternative mediators will be tested in separate single mediator models. Significance levels are set a priori at $p<0.05$.
ODI = Oswestry Disability Index

For peer review only - http://bmjopen.bmj.com/site/about/guidelines.xhtml

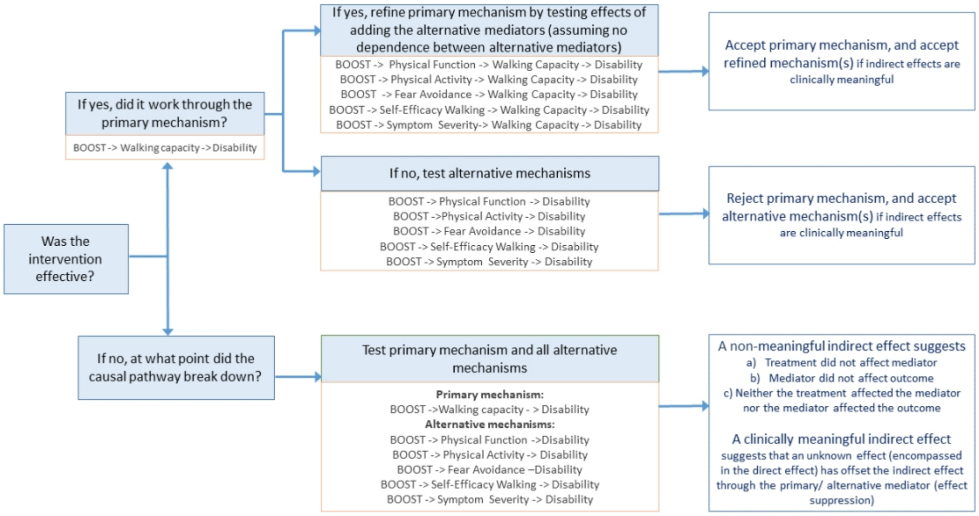

Heading for Figure 1:
Figure 1 Mediation protocol decision process

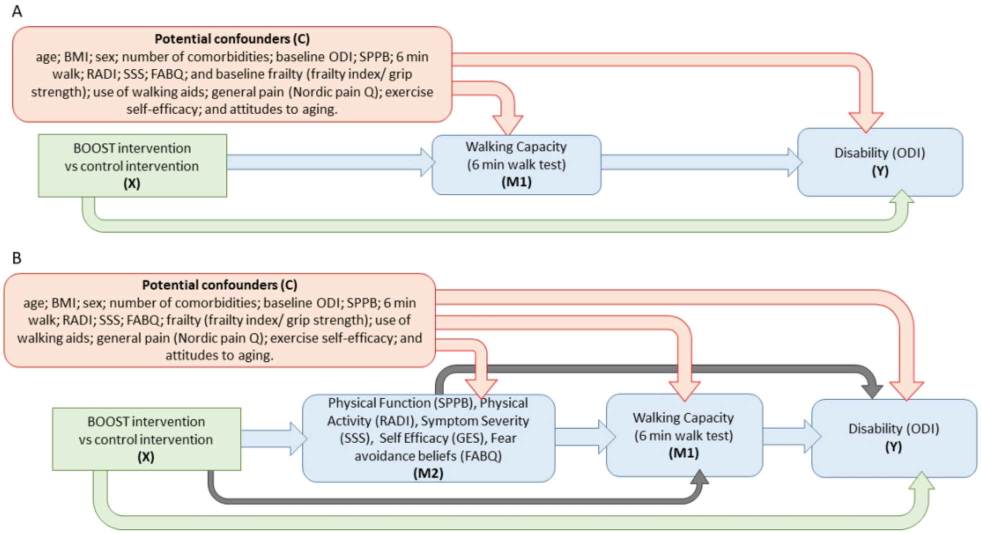

Heading for Figure 2: Figure 2 Figure 2 Directed acyclic graphics (DAG)

Legend for Figure 2: Blue lines represent indirect effects (mechanisms) of interest. Green lines represent direct effects (direct effect of treatment on outcome plus all unspecified indirect effects). Red lines represent possible effects that could induce confounding for indirect and direct effects. (A) A single mediator model where the intervention (X) exerts its effect on the outcome(s) Y, via an indirect path through the primary mediator (M1). (B) A serial multiple mediator model where the intervention (X) exerts its effect on the outcome (Y) via an indirect path through one of five alternative mediators (M2) and the primary mediator (M1), and a direct path (X to Y). ODI = Oswestry Disability Index, SPPB = S physical battery, RADI Rapid Assessment Disuse index, SSS = Swiss Spinal Stenosis Symptom severity scale, FABQ = fear avoidance beliefs questionnaire. M1 = 12 month measure, M2 = 6 month measures, Y = 12 month measure.

BMJ Open

Understanding the mechanisms of a combined physical and psychological intervention for people with neurogenic claudication: Protocol for a causal mediation analysis of the BOOST Trial

Journal:	*BMJ Open*
Manuscript ID	bmjopen-2020-037121.R2
Article Type:	Protocol
Date Submitted by the Author:	25-Apr-2020
Complete List of Authors:	Comer, Christine; Leeds Community Healthcare NHS Trust, Musculoskeletal and Rehabilitation Services; University of Leeds Faculty of Medicine and Health, Leeds Institute of Rheumatic and Musculoskeletal Medicine<br>Lee, Hopin; Hunter New England Population Health; University of Oxford Nuffield Department of Orthopaedics Rheumatology and Musculoskeletal Sciences<br>Williamson, Esther; Nuffield Department of Orthopaedics, Rheumatology and Musculoskeletal Sciences, University of Oxford, United Kingdom. , Centre for Rehabilitation Research in Oxford<br>Lamb, Sarah; University of Exeter, College of Medicine and Health; University of Oxford, NDORMS
**Primary Subject Heading**:	Health services research
Secondary Subject Heading:	Rehabilitation medicine
Keywords:	Spine < ORTHOPAEDIC & TRAUMA SURGERY, GERIATRIC MEDICINE, REHABILITATION MEDICINE

SCHOLARONE™
Manuscripts

I, the Submitting Author has the right to grant and does grant on behalf of all authors of the Work (as defined in the below author licence), an exclusive licence and/or a non-exclusive licence for contributions from authors who are: i) UK Crown employees; ii) where BMJ has agreed a CC-BY licence shall apply, and/or iii) in accordance with the terms applicable for US Federal Government officers or employees acting as part of their official duties; on a worldwide, perpetual, irrevocable, royalty-free basis to BMJ Publishing Group Ltd ("BMJ") its licensees and where the relevant Journal is co-owned by BMJ to the co-owners of the Journal, to publish the Work in this journal and any other BMJ products and to exploit all rights, as set out in our licence.

The Submitting Author accepts and understands that any supply made under these terms is made by BMJ to the Submitting Author unless you are acting as an employee on behalf of your employer or a postgraduate student of an affiliated institution which is paying any applicable article publishing charge ("APC") for Open Access articles. Where the Submitting Author wishes to make the Work available on an Open Access basis (and intends to pay the relevant APC), the terms of reuse of such Open Access shall be governed by a Creative Commons licence – details of these licences and which Creative Commons licence will apply to this Work are set out in our licence referred to above.

Other than as permitted in any relevant BMJ Author's Self Archiving Policies, I confirm this Work has not been accepted for publication elsewhere, is not being considered for publication elsewhere and does not duplicate material already published. I confirm all authors consent to publication of this Work and authorise the granting of this licence.

For peer review only

Understanding the mechanisms of a combined physical and psychological intervention for people with neurogenic claudication: Protocol for a causal mediation analysis of the BOOST Trial

Christine Comer [1,2], **Hopin Lee** [3,4] **Esther Williamson** [3] **and Sarah Lamb** [3,5]

1 Leeds Community Healthcare Musculoskeletal and Rehabilitation Services, Leeds Community Healthcare NHS Trust, Stockdale House, Victoria Road, Leeds UK

2 Leeds Institute of Rheumatic and Musculoskeletal Medicine, University of Leeds, Leeds, UK

3 Centre for Rehabilitation Research, Nuffield Department of Orthopaedics, Rheumatology and Musculoskeletal Sciences, University of Oxford, Oxford, UK

4 School of Medicine and Public Health, University of Newcastle, New South Wales, Australia

5 University of Exeter Medical School, Institute of Health Research, Devon, UK

Correspondence to Christine Comer, c.comer@leeds.ac.uk, +44 (0)113 3921521

Authors' contributions: all authors were involved in the design and write-up of this protocol.

Funding statement: Christine Comer is funded by a National Institute for Health Research (NIHR) Clinical Lectureship ICA-CL-2017-03-015 for this research project. This publication presents independent research. The views expressed are those of the author(s) and not necessarily those of the NHS, the NIHR or the Department of Health and Social Care.

Competing interests statement: None declared.

Word count 3,131

Keywords

Mediation, Causality, Spinal Stenosis, Behaviour and behaviour mechanisms, Exercise, Rehabilitation

For peer review only - http://bmjopen.bmj.com/site/about/guidelines.xhtml

ABSTRACT

Introduction
Conservative treatments such as exercise are recommended for the management of people with neurogenic claudication from spinal stenosis. However, the effectiveness and mechanisms of effect are unknown. This protocol outlines an a-priori plan for a secondary analysis of a multi-centre randomised controlled trial of a physiotherapist-delivered, combined physical and psychological intervention (Better Outcomes for Older people with Spinal Trouble (BOOST) programme).

Methods and analyses
We will use causal mediation analysis to estimate the mechanistic effects of the BOOST programme on the primary outcome of disability (measured by the Oswestry Disability Index). The primary mechanism of interest is walking capacity, and secondary mediators include fear avoidance behaviour, walking self-efficacy, physical function, physical activity and/or symptom severity. All mediators will be measured at 6 months and the outcome will be measured at 12 months from randomisation. Patient characteristics and possible confounders of the mediator-outcome effect will be measured at baseline. Sensitivity analyses will be conducted to evaluate the robustness of the estimated effects to varying levels of residual confounding.

Ethics and Dissemination
Ethical approval was given on the 03 March 2016 (National Research Ethics Committee number: 16/LO/0349). The results of this analysis will be disseminated in peer-reviewed journals and at relevant scientific conferences.

Trial Registration number ISRCTN12698674

Strengths and Limitations of the Study
- Causal mediation analysis of clinical trials can explain how an effective intervention works, or why an ineffective intervention does not work.
- This protocol describes a planned analysis of the underlying causal mechanisms of the BOOST rehabilitation programme; a combined physical and psychological intervention for people with neurogenic claudication symptoms.
- We propose exploratory analysis of several potential causal pathways based on theoretical mechanisms of action, acknowledging the risks associated with multiple testing.
- The proposed models will estimate the extent to which candidate mediators (walking capacity, fear avoidance behaviour, walking self-efficacy, physical function, physical activity, and symptom severity) mediate effects on disability measured by the Oswestry Disability index.

For peer review only - http://bmjopen.bmj.com/site/about/guidelines.xhtml

INTRODUCTION

Neurogenic claudication (NC) is a leading cause of disability and lost independence in older adults [1-3]. NC is the term used to describe the typical symptoms associated with lumbar spinal stenosis (LSS) [4]. These symptoms are provoked by walking, and include pain, aching, paraesthesia and fatigue which radiate from the spine into the buttocks and legs [5]. NC often leads to reduced mobility [6] contributing to disability, frailty and falls [7-12], increased risks of comorbidity, social isolation and loss of independence [13, 14]. The symptoms are thought to arise because of reduced space and subsequent compromise of the neural and vascular elements in the lumbar spine [4] resulting from degenerative changes in the aging spine [15].

LSS has become the most common reason for spinal surgery in people over 65 years of age [16]. Because of the risks, costs and unclear benefits associated with surgical treatment [16-18], it is widely accepted that non-surgical treatment should be first line care [19, 20]. Advice, exercise and self-management strategies are often used in clinical practice [21-23] but there is limited robust evidence to demonstrate their efficacy and effectiveness [17].

The BOOST trial is a large multicentre RCT testing the effectiveness of a 12-week combined physical and psychological intervention delivered by physiotherapists in a group setting. The primary focus of this randomised trial is to estimate the effect of the BOOST intervention on patient-reported disability. There is, however, growing recognition of the importance of evaluating hypothesised treatment mechanisms in randomised trials [24]. Doing so can explain how an effective intervention works, or why an ineffective intervention does not work.

Objectives

We present an a priori protocol for a secondary analysis of the BOOST trial to evaluate the effects on the primary mediator of a change in walking capacity, and to explore alternative mediators of changes in fear avoidance behaviour, walking self-efficacy, physical function, physical activity, and symptom severity. The aim of this planned analysis is to contribute to our understanding of treatment mechanisms and provide valuable information to guide future intervention development and implementation.

METHODS AND ANALYSES

Design

This study is a causal mediation analysis of the National Institute of Health Research (NIHR) funded BOOST trial. The BOOST trial [ISRCTN12698674] is a high-quality multi-centred randomised controlled trial. It aims to evaluate the clinical- and cost-effectiveness of a physiotherapist-delivered, combined physical and psychological intervention for community-dwelling older adults with symptoms of NC, compared to

For peer review only - http://bmjopen.bmj.com/site/about/guidelines.xhtml

a control treatment of best practice (standardised advice and education). BOOST trial details are published separately in the trial protocol [25].

Patient and public involvement

Patient and public involvement has been embedded in the BOOST trial and is described fully in the trial protocol [25]

Participants

438 participants with symptoms of NC from across 15 primary- and secondary- care centres in the UK have been randomised to the trial intervention or control treatment at a 2:1 ratio. We recruited participants over the age of 65 years from spinal clinics within primary and secondary care centres (n=394) and via a primary care survey of community dwelling older adults (n=44). They were eligible for the study if they presented with symptoms consistent with NC (back and/or leg pain or symptoms aggravated by standing or walking and eased by sitting or bending) and were able to participate safely in the group rehabilitation programme.

BOOST trial Intervention

A detailed description of the BOOST intervention and its development have been published elsewhere [26]. In summary, the BOOST intervention combines physical and psychological components in a programme delivered to small groups of participants over twelve sessions. The physical component comprises a 60-minute exercise programme at each session, including exercises for strength, balance and flexibility, and a walking circuit. The level of exercise difficulty is individually tailored, and is progressed over the 12-week programme by increasing the number of repetitions, sets, and increasing weights and/ or speed. The psychological component consists of a series of 30-minute discussions at each of the twelve sessions. Early sessions are aimed at facilitating self-efficacy and safety, including addressing unhelpful pain behaviour and fear-related avoidance of movement; providing simple concepts of exercise physiology relevant to improving mobility; and introducing basic behavioural techniques of pacing and graded activity. Subsequent sessions use peer-support to encourage exercise adherence, identify facilitators and barriers, and reinforce long-term exercise adherence through peer discussion about exercise confidence and motivation, access to local activities, and advice on coping with acute symptom flare-ups. Over the 12-week supervised programme, session frequency reduces from twice weekly to once weekly and then to once fortnightly. Concurrently, home exercise programmes are introduced with the aim of encouraging participants to increase their level of independent, unsupervised exercise and physical activity at home.

We developed the intervention collaboratively with clinical experts and patients to address some of the unique challenges of managing back problems in older people. These include age-related physical changes of reduced muscle strength and fitness, in addition to psychological factors such as depression [27] and unhelpful behaviours and beliefs about aging and exercise which are known to negatively impact on outcomes and act as barriers to accessing and engaging with treatment [28-31].

Control intervention

For peer review only - http://bmjopen.bmj.com/site/about/guidelines.xhtml

The control intervention consists of a one-hour individual session with a physiotherapist for assessment, advice and education about NC and being physically active, advice on exercises including flexion-based exercises, use of medications and when to seek more advice. The advice and education are reinforced in a leaflet and a further two review appointments are available if required.

Outcomes

The Oswestry Disability Index (ODI) [32] is the primary outcome measure for the BOOST Trial. The ODI (also known as the Oswestry Low Back Pain Disability Questionnaire) is the most widely used measure in low back pain research and is considered the 'gold standard' of low back functional outcome tools. It is commonly used to provide a patient reported measure of walking limitation and other functional limitations in Lumbar spinal stenosis trials, alongside objective measures of walking ability [33].

The ODI comprises a series of questions about how pain impacts on the patient's ability to carry out a range of daily functions. For each question, possible answers are scored on a scale of 0 to 5. These scores are summed and expressed as a percentage of the maximum possible score for disability, with higher scores representing worse disability. In the BOOST trial, ODI scores are measured for each participant at baseline, and at 6 and 12 months after randomisation.

Important secondary outcome measures for the BOOST trial focus on walking capacity, and on fear avoidance behaviour, walking self-efficacy, physical function, physical activity and symptom severity: The six-minute walk test provides an objective measure of walking capacity and entails a timed measure of walking distance which is easily performed in the clinical setting [34]. The Short Physical Performance Battery (SPPB) provides an objective measure of physical performance which incorporates a balance test, a functional lower limb strength test of 5 timed chair stands, and a timed walking test over 8 feet. This is similarly easy to complete in a clinic [35]. Outcome questionnaires completed by participants provide subjective evaluation of relevant constructs. These include physical activity measured by a modified version of the Rapid Assessment Disuse Index (RADI) [36]; severity of stenotic symptoms measured by the Swiss Spinal Stenosis Questionnaire (SSS) symptom severity subscale [37]; walking self-efficacy measured by a single item from the Modified Gait Efficacy Scale (GES) [38]; and fear of movement measured by the physical activity subscale of the Fear Avoidance Beliefs Questionnaire (FABQ) [39]. These outcomes are all measured at baseline, and at 6 and 12 months following randomisation.

Causal Mediation Analysis - Rationale

We will test the mechanisms of the BOOST intervention for patients with NC by estimating the extent to which different potential mediators explain the effect of the BOOST intervention on participants' ODI outcome scores. This will be achieved by deconstructing the total effect of the BOOST treatment into indirect and direct effects. The indirect effect is the path through which the intervention affects the outcome via a selected mediator. The direct effect is the remaining total effect that is not encompassed by the indirect effect.

For peer review only - http://bmjopen.bmj.com/site/about/guidelines.xhtml

Walking capacity has been selected as the primary mediator for our causal mediation analysis model. Walking limitation is a hallmark of disability in LSS [40] and a serious concern for people with LSS who often seek healthcare because of reduced mobility [41-43]. The BOOST intervention incorporates both physical and cognitive behavioural components aimed at reducing disability, and increased walking is a key target.

The BOOST intervention is underpinned by a strong theoretical premise for addressing age-related decline in mobility. Exercise components of the intervention aim to improve physical function by targeting reduced muscle strength, balance and sensory impairments [44-53]. Whilst cognitive behavioural approaches have not been well investigated in people with LSS [54], this is an important component of the BOOST intervention based on evidence that addressing unhelpful beliefs and behaviours can mediate changes in both disability and symptom severity in low back pain populations [55-60]. In addition, self-efficacy is likely to play a role in mediating disability, physical function and activity in older adults with chronic musculoskeletal conditions [61-63]. Alternative mediators that we will include in our causal mediation models therefore include physical function (SPPB), physical activity (RADI), fear avoidance behaviour (FABQ), and walking self-efficacy (GES).

Our final alternative mediator is symptom severity (SSS). Little is known about how exercise affects NC symptom severity [64]. In theory, better muscular strength, control and endurance, and increased flexibility are likely not only to enhance physical ability [65, 66], but also to reduce pain by altering the loading of the musculoskeletal system and changing central pain processes and immune system responses [67-69]. Neuroischaemic lower limb symptoms such as paraesthesia, numbness, sensory and balance changes might also be improved through reduced nerve root ischaemia and venous pooling as a result of exercise [4, 70].

To understand the mechanisms of the BOOST intervention, we plan to test our primary hypothesis that changes in disability will be mediated primarily by changes in walking capacity, and that various mechanisms might explain these changes in walking capacity: We hypothesise that the progressive exercise component of the BOOST intervention will exert its effect on walking capacity via initial changes in physical function and physical activity levels during treatment. We further hypothesise that the cognitive behavioural components of the intervention will exert effects on walking capacity through reductions in fear avoidance behaviour and increased self-efficacy. There is also the potential for both the exercise and the cognitive behavioural components of the intervention to reduce symptom severity, resulting in increased walking capacity.

Our secondary hypothesis is that the causal effects of the BOOST intervention on disability will be explained through changes in fear avoidance behaviour, self-efficacy, physical function, physical activity and/or symptom severity (SSS) without exerting any effects on walking capacity.

If the BOOST intervention is found to be effective, causal mediation analysis will explain how the intervention works. Conversely, if the intervention is not found to be effective, causal mediation analysis will identify where the hypothesised mechanism broke down [71].

For peer review only - http://bmjopen.bmj.com/site/about/guidelines.xhtml

Causal Mediation analysis - models

The decision-making process that will guide our analyses is laid out in Figure 1. Table 1 provides an overview of the planned mediation models contingent on whether or not the BOOST intervention is effective:

► If the intervention is effective, our primary objective is to estimate the extent to which the joint effects of walking capacity, fear avoidance behaviour, walking self-efficacy, physical function, physical activity, and symptom severity mediate this effect.

Our secondary objective will be to further refine this model through the candidate primary mediator, walking capacity; and via five secondary mediators (fear avoidance behaviour, walking self-efficacy, physical function, physical activity, and symptom severity). If the indirect effect through the primary mediator is significant, we will consider sequentially ordered multiple mediator models through secondary mediators. The serial multiple mediator models accounting for potential confounders are presented in the Directed Acyclic Graphics (DAGs) in Figure 2.

► If the intervention is not effective, our primary objective is to determine where the causal paths break down. All potential mediators (walking capacity, fear avoidance, self-efficacy, physical function, physical activity and symptom severity) will be evaluated.

If alternative pathways of mediation emerge during the analysis of the trial and associated qualitative data these will also be investigated. The publication of this protocol does not preclude other analyses if indicated.

Statistical Analysis

We will use a model-based inference approach for causal mediation analysis [72]. All analyses will be conducted in R (The R Foundation for Statistical Computing) using the "*medflex*" and "*mediation*" packages [73, 74].

To estimate a joint indirect effect, we will consider a natural indirect effect of the intervention on the outcome that is exerted through a vector of all six mediators (walking capacity, fear avoidance behaviour, walking self-efficacy, physical function, physical activity, and symptom severity). The advantage of this approach is that we can consider all possible mechanisms simultaneously and relax the assumption of omitting confounders of the mediator-outcome effect that is affected by randomisation. We will use the imputation-based approach outlined by Steen et al [75, 76] to fit a natural effect model with robust standard errors based on the sandwich-estimator – the recommended approach when the ordering of multiple mediators is unknown.

For peer review only

To consider the mediators independently, we will construct single mediator models for the ODI outcome. For each single mediator model (primary mediator model of walking capacity, and secondary mediator models of fear avoidance, self-efficacy, physical function, physical activity and symptom severity), we will estimate the intervention-mediator effect, the mediator-outcome effect, the average causal mediation effect (ACME), average direct effect (ADE) and the average total effect (ATE). We will also estimate the proportion mediated in each single mediator model. The ACME is the average intervention effect through the mediator; ADE is the average intervention effect that works through all other mechanism excluding the selected mediator; and the ATE is the average effect of the intervention on the outcome. The ATE is the sum of the ACME and ADE on the additive scale. The proportion mediated is the fraction of the ATE that is explained by the ACME.

We will fit two regression models: the mediator model, and the outcome model. The mediator model will use linear regression with treatment allocation as the independent variable, and the mediator as the dependent variable, with the baseline level of the mediator as a covariate. The outcome model for disability will also use linear regression. Each outcome model will be constructed with the mediator as the independent variable, the outcome as the dependent variable, and treatment allocation, baseline values of the meditator and outcome variable in addition to the set of observed pre-treatment confounders as covariates. Because the mediators are not randomised, it is possible for the mediator-outcome effects to be confounded. To address this problem, we will adjust for the effects of pre-treatment confounders based on theorised causal relationships with the mediators and outcome variables from expert clinical knowledge and available evidence. These potential confounders include age, BMI, sex, number of comorbidities, in addition to baseline measures of disability, physical function, walking capacity, physical activity, symptom severity and fear-avoidance behaviour, and baseline frailty (frailty index/ grip strength); use of walking aids; general pain (Nordic pain Q); exercise self-efficacy; and attitudes to aging. We will also conduct a sensitivity analysis to determine the robustness of the estimated ACME and ADE to possible unmeasured confounding [77]. We will also include an interaction term (treatment allocation X mediator) in the outcome models to allow for an intervention-mediator interaction effect on the outcome and to increase model flexibility [78].

Using mediator and outcome regression models, we will simulate potential values of the mediator for each participant under each level of the intervention; then simulate potential outcome values for each participant under all combinations of the intervention and simulated mediator values. From these observed and simulated potential values of the mediator and outcome, we will calculate point estimates for the ACME, ADE, and TE and their 95% confidence intervals using 1000 bootstrapped simulations with the percentile method.

For multiple mediator models, we will use an expanded framework. [79] Multiple mediator models will only be constructed if the alternative mediator (fear avoidance, self-efficacy, physical function, physical activity and symptom severity) and primary mediator (walking capacity) are associated [79]. We will use the *multimed* function from the *mediation* package to estimate the ACME and ADE, and the sensitivity parameters.

For peer review only - http://bmjopen.bmj.com/site/about/guidelines.xhtml

We will assess the proportion of missing mediator and outcome data. We will conduct all analyses on complete cases if the proportion of missing data is <5%. If missing data exceeds 5% we will use multiple imputation by chain equations to impute 10 datasets using the "mice" package [80].

To facilitate clinical interpretation, we will calculate how much change in a given mediator would equate to a minimally clinically important difference (MCID) of disability (5 point change in ODI score [81]) by using the regression coefficients from the outcome model, we will divide the MCID by the mediator coefficient and 95% Confidence Interval from the outcome regression model.

Clinical Implications

We do not currently know whether non-surgical interventions are effective in reducing disability reported by older people with NC symptoms. The BOOST trial will provide important high-quality evidence about the effectiveness of an intervention incorporating components to address both physical and psychological impairments. The targets of this comprehensive intervention include walking capacity, physical function, physical activity, self-efficacy, unhelpful beliefs about activity, and symptom severity. We cannot assume, however, that if the intervention improves any of these variables the participants will experience benefits in patient-reported outcomes such as the ODI.

Mediation analysis conducted in parallel to an RCT can address specific questions about the underlying mechanisms of the intervention being tested. This protocol describes the planned causal mediation analysis that we will undertake to evaluate hypothetical mechanisms that underlie the effect of the BOOST rehabilitation programme. This will help us to understand how the BOOST intervention works, or doesn't work.

If the BOOST intervention is found to be effective and this mediation analysis indicates that improvements in ODI scores are mediated by walking capacity, which in turn is mediated by improved self-efficacy, then this provides valuable information for refining the intervention with greater focus on self-efficacy to optimise treatment effectiveness. If, on the other hand, the BOOST trial is not found to be produce a significant improvement in patient reported disability, then the analysis will help us to understand where the hypothesises causal mechanisms of treatment break down. For example, we may find that although the BOOST intervention improves walking capacity, this does not translate into an improvement in ODI score and may therefore not be an appropriate treatment target. The causal analysis will therefore provide valuable information for designing, refining and implementing an effective intervention.

Because this is an exploratory study we have selected several mediators based on our current clinical understanding and evidence for exercise and behavioural treatments. Several potential causal pathways will therefore be explored, and we acknowledge the risks associated with this multiple testing. If alternative pathways of mediation emerge during the analysis of the trial and associated qualitative data, we will investigate these further. The publication of this protocol does not preclude other

For peer review only - http://bmjopen.bmj.com/site/about/guidelines.xhtml

analyses if indicated. Future studies may be needed to further explore the inter-relationship of the mediators explored in our analyses.

Conclusion

Clinical trials do not address questions around *how* an intervention affects outcomes, and therefore may not identify the most effective treatment targets for a particular patient population. Mediation analysis of clinical trials can estimate how much the total effect of the treatment can be attributed to pre-specified treatment targets. Improving our understanding of these treatment mechanisms can generate evidence that can be used to tailor and refine treatments and optimise clinical effectiveness. In this study, we will estimate the causal mediation effects of a rehabilitation programme incorporating exercise and behavioural components for patients with NC.

Ethics: Ethical approval was given on the 03 March 2016 (National Research Ethics Committee number: 16/LO/0349). The results of this analysis will be disseminated in peer-reviewed journals and at relevant scientific conferences.

Acknowledgements: We thank Judith Fitch, lead PPI representative and a co-applicant who contributed to the design of the primary trial, and the patient and public involvement representatives who have provided advice and feedback on the development of the BOOST intervention, patient materials and conduct of the trial.

Authors' contributions: CC, HL, EW and SL conceived and designed the study. SL and EW led the primary trial and data acquisition. SL is the Chief Investigator. HL, EW, CC, SL contributed to the analytical plan. CC led the preparation of the draft and HL, EW, SL edited and contributed to the final manuscript.

Funding statement: Christine Comer is funded by a National Institute for Health Research (NIHR) Clinical Lectureship ICA-CL-2017-03-015 for this research project. This publication presents independent research. The views expressed are those of the author(s) and not necessarily those of the NHS, the NIHR or the Department of Health and Social Care.

Competing interests statement: None declared.

For peer review only - http://bmjopen.bmj.com/site/about/guidelines.xhtml

REFERENCES

1. Ishimoto, Y., et al., *Prevalence of symptomatic lumbar spinal stenosis and its association with physical performance in a population-based cohort in Japan: the Wakayama Spine Study.* Osteoarthritis Cartilage, 2012. **20**(10): p. 1103-8.
2. Fanuele, J.C., et al., *The impact of spinal problems on the health status of patients: have we underestimated the effect?* Spine (Phila Pa 1976), 2000. **25**(12): p. 1509-14.
3. Winter, C.C., et al., *Walking ability during daily life in patients with osteoarthritis of the knee or the hip and lumbar spinal stenosis: a cross sectional study.* BMC Musculoskelet Disord, 2010. **11**: p. 233.
4. Lurie, J. and C. Tomkins-Lane, *Management of lumbar spinal stenosis.* BMJ, 2016. **352**: p. h6234.
5. Kreiner, D., et al., *Evidence-Based Clinical Guidelines for Multidisciplinary Spine Care.* 2011: p. 43-44.
6. Ammendolia, C., et al., *What interventions improve walking ability in neurogenic claudication with lumbar spinal stenosis? A systematic review.* 2014. **23**(6): p. 1282-1301.
7. Eggermont, L.H., R.H. Shmerling, and S.G. Leveille, *Tender point count, pain, and mobility in the older population: the mobilize Boston study.* The Journal of Pain, 2010. **11**(1): p. 62-70.
8. Koponen, M.P., et al., *Analgesic use and frailty among community-dwelling older people.* Drugs & aging, 2013. **30**(2): p. 129-136.
9. Leveille, S.G., et al., *The pathway from musculoskeletal pain to mobility difficulty in older disabled women.* Pain, 2007. **128**(1-2): p. 69-77.
10. Leveille, S.G., et al., *Chronic musculoskeletal pain and the occurrence of falls in an older population.* Jama, 2009. **302**(20): p. 2214-2221.
11. Makris, U.E., et al., *Restricting Back Pain and Subsequent Mobility Disability in Community-Living Older Persons.* Journal of the American Geriatrics Society, 2014. **62**(11): p. 2142-2147.
12. Shega, J.W., et al., *Persistent pain and frailty: a case for homeostenosis.* Journal of the American Geriatrics Society, 2012. **60**(1): p. 113-117.
13. Fried, L.P., et al., *Frailty in older adults: evidence for a phenotype.* The Journals of Gerontology Series A: Biological Sciences and Medical Sciences, 2001. **56**(3): p. M146-M157.
14. Troutman-Jordan, M. and J. Staples, *Successful aging from the viewpoint of older adults.* Res Theory Nurs Pract, 2014. **28**(1): p. 87-104.
15. Chad, D.A.J.N.c., *Lumbar spinal stenosis.* 2007. **25**(2): p. 407-418.
16. Deyo, R.A., et al., *Trends, major medical complications, and charges associated with surgery for lumbar spinal stenosis in older adults.* JAMA, 2010. **303**(13): p. 1259-65.
17. Zaina, F., et al., *Surgical Versus Nonsurgical Treatment for Lumbar Spinal Stenosis.* Spine, 2016. **41**(14): p. E857-E868.
18. Kovacs, F.M., G. Urrutia, and J.D. Alarcon, *Surgery versus conservative treatment for symptomatic lumbar spinal stenosis: a systematic review of randomized controlled trials.* Spine (Phila Pa 1976), 2011. **36**(20): p. E1335-51.
19. Ammendolia, C., et al., *Nonoperative treatment for lumbar spinal stenosis with neurogenic claudication.* Cochrane Database of Systematic Reviews, 2013(8): p. 62.
20. Rousing, R., et al., *Danish national clinical guidelines for surgical and nonsurgical treatment of patients with lumbar spinal stenosis.* Eur Spine J, 2019. **28**(6): p. 1386-1396.
21. Balakatounis, K.C.P., Kalomoira A; Mitsiokapa, Evanthia A; Mavrogenis, Angoules, Antonios G; Papthanasiou, Jannis; Papgelopoulos, Panayiotis J, *Evidence-Based Evaluation and Current Practice of Non-Operative Treatment Strategies for Lumbar Stenosis*

Folia Medica, 2011. **53**(3): p. 5-14.

For peer review only - http://bmjopen.bmj.com/site/about/guidelines.xhtml

22. Comer, C.M., et al., *Assessment and management of neurogenic claudication associated with lumbar spinal stenosis in a UK primary care musculoskeletal service: a survey of current practice among physiotherapists.* BMC musculoskeletal disorders, 2009. **10**(1): p. 121.
23. Matsuwaka, S.T., B.C.J.C.P.M. Liem, and R. Reports, *The Role of Exercise in Treatment of Lumbar Spinal Stenosis Symptoms.* 2018. **6**(1): p. 36-44.
24. Mansell, G., et al., *How can we design low back pain intervention studies to better explain the effects of treatment?* 2014, LWW.
25. Williamson, E., et al., *Better Outcomes for Older people with Spinal Trouble (BOOST) Trial: a randomised controlled trial of a combined physical and psychological intervention for older adults with neurogenic claudication, a protocol.* 2018. **8**(10): p. e022205.
26. Ward, L., et al., *Development and delivery of the BOOST (Better Outcomes for Older adults with Spinal Trouble) intervention for older adults with neurogenic claudication.* 2019.
27. McKillop, A.B., L.J. Carroll, and M.C. Battié, *Depression as a prognostic factor of lumbar spinal stenosis: a systematic review.* The Spine Journal, 2014. **14**(5): p. 837-846.
28. Lamb, S., et al., *A multicentred randomised controlled trial of a primary care-based cognitive behavioural programme for low back pain. The Back Skills Training (BeST) trial.* Health Technol Assess, 2010. **14**(41): p. 1-253.
29. Makris, U.E., et al., *Ageism, negative attitudes, and competing co-morbidities–why older adults may not seek care for restricting back pain: a qualitative study.* BMC geriatrics, 2015. **15**(1): p. 39.
30. Thielke, S., J. Sale, and M.C. Reid, *Aging: are these 4 pain myths complicating care?* The Journal of family practice, 2012. **61**(11): p. 666.
31. Wood, D.W., A.J. Haig, and K.S. Yamakawa, *Fear of movement/(re) injury and activity avoidance in persons with neurogenic versus vascular claudication.* The Spine Journal, 2012. **12**(4): p. 292-300.
32. Fairbank, J.C.T. and P.B. Pynsent, *The Oswestry Disability Index.* Spine, 2000. **25**(22): p. 2940-2952.
33. Jespersen, A.B. and M. Gustafsson, *Correlation between the Oswestry Disability Index and objective measurements of walking capacity and performance in patients with lumbar spinal stenosis: a systematic literature review.* Eur Spine J, 2018. **27**(7): p. 1604-1613.
34. Harada, N.D., V. Chiu, and A.L. Stewart, *Mobility-related function in older adults: assessment with a 6-minute walk test.* Arch Phys Med Rehabil, 1999. **80**(7): p. 837-41.
35. Guralnik, J.M., et al., *Lower-extremity function in persons over the age of 70 years as a predictor of subsequent disability.* N Engl J Med, 1995. **332**(9): p. 556-61.
36. Shuval, K., et al., *Sedentary behaviour and physical inactivity assessment in primary care: the Rapid Assessment Disuse Index (RADI) study.* 2014. **48**(3): p. 250-255.
37. Stucki, G., et al., *Measurement properties of a self-administered outcome measure in lumbar spinal stenosis.* Spine, 1996. **21**(7): p. 796-803.
38. Newell, A.M., et al., *The modified gait efficacy scale: establishing the psychometric properties in older adults.* 2012. **92**(2): p. 318-328.
39. Waddell, G., et al., *A Fear-Avoidance Beliefs Questionnaire (FABQ) and the role of fear-avoidance beliefs in chronic low back pain and disability.* Pain, 1993. **52**(2): p. 157-68.
40. Conway, J., C.C. Tomkins, and A.J. Haig, *Walking assessment in people with lumbar spinal stenosis: capacity, performance, and self-report measures.* The Spine Journal, 2011. **11**(9): p. 816-823.
41. Amundsen, T., et al., *Lumbar spinal stenosis. Clinical and radiologic features.* 1995. **20**(10): p. 1178-1186.
42. Yamashita, K., et al., *Correlation of patient satisfaction with symptom severity and walking ability after surgical treatment for degenerative lumbar spinal stenosis.* Spine, 2003. **28**(21): p. 2477-2481.

For peer review only - http://bmjopen.bmj.com/site/about/guidelines.xhtml

43. Markman, J.D., et al., *Evaluation of outcome measures for neurogenic claudication: A patient-centered approach.* Neurology, 2015. **85**(14): p. 1250-6.
44. Rantakokko, M., et al., *Mobility decline in old age.* 2013. **41**(1): p. 19-25.
45. Keysor, J.J., *Does late-life physical activity or exercise prevent or minimize disablement? A critical review of the scientific evidence.* Am J Prev Med, 2003. **25**(3 Suppl 2): p. 129-36.
46. Andrews, J.S., et al., *Muscle strength, muscle mass, and physical disability in women with systemic lupus erythematosus.* Arthritis Care Res (Hoboken), 2015. **67**(1): p. 120-7.
47. Clemson, L., et al., *Integration of balance and strength training into daily life activity to reduce rate of falls in older people (the LiFE study): randomised parallel trial.* BMJ, 2012. **345**: p. e4547.
48. Hayden, J., et al., *Exercise therapy for treatment of non-specific low back pain.* Cochrane Database of Systematic Reviews, 2005(3).
49. Daniels, R., et al., *Interventions to prevent disability in frail community-dwelling elderly: a systematic review.* BMC Health Serv Res, 2008. **8**: p. 278.
50. Rantanen, T., et al., *Disability, physical activity, and muscle strength in older women: the Women's Health and Aging Study.* Archives of physical medicine and rehabilitation, 1999. **80**(2): p. 130-135.
51. Van Heuvelen, M.J., et al., *Physical fitness related to disability in older persons.* Gerontology, 2000. **46**(6): p. 333-341.
52. REJC, E., A. DEL TORTO, and S.J.A.K. LAZZER, *BENEFITS OF AEROBIC EXERCISE TRAINING WITH RECOMMENDATIONS FOR HEALTHY AGING.* 2017. **8**(2).
53. Lee, P.G., E.A. Jackson, and C.R.J.A.f.p. Richardson, *Exercise Prescriptions in Older Adults.* 2017. **95**(7).
54. Richmond, H., et al., *The Effectiveness of Cognitive Behavioural Treatment for Non-Specific Low Back Pain: A Systematic Review and Meta-Analysis.* PLoS One, 2015. **10**(8): p. e0134192.
55. Eccleston, C., A.C. Williams, and S. Morley, *Psychological therapies for the management of chronic pain (excluding headache) in adults.* Cochrane Database Syst Rev, 2009(2): p. CD007407.
56. Hoffman, B.M., et al., *Meta-analysis of psychological interventions for chronic low back pain.* Health Psychol, 2007. **26**(1): p. 1-9.
57. Wertli, M.M., et al., *Fear-avoidance beliefs—a moderator of treatment efficacy in patients with low back pain: a systematic review.* The Spine Journal, 2014. **14**(11): p. 2658-2678.
58. Mansell, G., et al., *Mediators of Treatment Effect in the Back In Action Trial: Using Latent Growth Modeling to Take Change Over Time Into Account.* Clin J Pain, 2017. **33**(9): p. 811-819.
59. Collins, T.C., S. Lunos, and J.S. Ahluwalia, *Self-efficacy is associated with walking ability in persons with diabetes mellitus and peripheral arterial disease.* Vasc Med, 2010. **15**(3): p. 189-95.
60. Danks, K.A., et al., *Relationship Between Walking Capacity, Biopsychosocial Factors, Self-efficacy, and Walking Activity in Persons Poststroke.* J Neurol Phys Ther, 2016. **40**(4): p. 232-8.
61. Karasawa, Y., et al., *Association between change in self-efficacy and reduction in disability among patients with chronic pain.* 2019. **14**(4): p. e0215404.
62. Focht, B.C., et al., *Exercise, self-efficacy, and mobility performance in overweight and obese older adults with knee osteoarthritis.* 2005. **53**(5): p. 659-665.
63. Seymour, R.B., et al., *Comparison of two methods of conducting the Fit and Strong! program.* 2009. **61**(7): p. 876-884.
64. Ammendolia, C., et al., *Nonoperative treatment for lumbar spinal stenosis with neurogenic claudication.* Cochrane Database Syst Rev, 2013(8): p. CD010712.

For peer review only - http://bmjopen.bmj.com/site/about/guidelines.xhtml

65. Whitman, J.M., et al., *A comparison between two physical therapy treatment programs for patients with lumbar spinal stenosis: a randomized clinical trial.* Spine (Phila Pa 1976), 2006. **31**(22): p. 2541-9.
66. Vincent, H.K., et al., *Resistance exercise, disability, and pain catastrophizing in obese adults with back pain.* Medicine and science in sports and exercise, 2014. **46**(9): p. 1693.
67. Booth, J., et al., *Exercise for chronic musculoskeletal pain: A biopsychosocial approach.* Musculoskeletal Care, 2017. **15**(4): p. 413-421.
68. Smith, B.E., et al., *Musculoskeletal pain and exercise-challenging existing paradigms and introducing new.* Br J Sports Med, 2018.
69. Runhaar, J., et al., *Identifying potential working mechanisms behind the positive effects of exercise therapy on pain and function in osteoarthritis; a systematic review.* Osteoarthritis Cartilage, 2015. **23**(7): p. 1071-82.
70. Tomkins, C.C., et al., *Physical therapy treatment options for lumbar spinal stenosis.* J Back Musculoskelet Rehabil, 2010. **23**(1): p. 31-7.
71. Imai, K., et al., *Unpacking the black box of causality: Learning about causal mechanisms from experimental and observational studies.* 2011. **105**(4): p. 765-789.
72. Imai, K., L. Keele, and D.J.P.m. Tingley, *A general approach to causal mediation analysis.* 2010. **15**(4): p. 309.
73. Tingley, D., et al., *Mediation: R package for causal mediation analysis.* 2014.
74. Steen, J., et al., *Medflex: an R package for flexible mediation analysis using natural effect models.* 2017. **76**(11).
75. Steen, J., et al. *Medflex: flexible mediation analysis using natural effect models in R*. in *UK Causal Inference Meeting*. 2015.
76. VanderWeele, T. and S.J.E.m. Vansteelandt, *Mediation analysis with multiple mediators.* 2014. **2**(1): p. 95-115.
77. Imai, K., L. Keele, and T.J.S.s. Yamamoto, *Identification, inference and sensitivity analysis for causal mediation effects.* 2010: p. 51-71.
78. VanderWeele, T., *Explanation in causal inference: methods for mediation and interaction*. 2015: Oxford University Press.
79. Imai, K. and T.J.P.A. Yamamoto, *Identification and sensitivity analysis for multiple causal mechanisms: Revisiting evidence from framing experiments.* 2013. **21**(2): p. 141-171.
80. Buuren, S.v. and K.J.J.o.s.s. Groothuis-Oudshoorn, *mice: Multivariate imputation by chained equations in R.* 2010: p. 1-68.
81. Cleland, J.A., et al., *Psychometric properties of selected tests in patients with lumbar spinal stenosis.* 2012. **12**(10): p. 921-931.

For peer review only - http://bmjopen.bmj.com/site/about/guidelines.xhtml

Figure 1 Mediation protocol decision process

Figure 2 Directed acyclic graphics (DAG)

Legend for Figure 2

Blue lines represent indirect effects (mechanisms) of interest. Green lines represent direct effects (direct effect of treatment on outcome plus all unspecified indirect effects). Red lines represent possible effects that could induce confounding for indirect and direct effects. (A) A single mediator model where the intervention (X) exerts its effect on the outcome(s) Y, via an indirect path through the primary mediator (M1). (B) A serial multiple mediator model where the intervention (X) exerts its effect on the outcome (Y) via an indirect path through one of five alternative mediators (M2) and the primary mediator (M1), and a direct path (X to Y). ODI = Oswestry Disability Index, SPPB = S physical battery, RADI Rapid Assessment Disuse index, SSS = Swiss Spinal Stenosis Symptom severity scale, FABQ = fear avoidance beliefs questionnaire. M1 = 12 month measure, M2 = 6 month measures, Y = 12 month measure.

For peer review only

Table 1. Overview of Mediator Models

Model	Treatment	Alternative mediators at 6 months (M2)	Primary mediator at 6 months (M1)	Outcome at 12 months
If the total effect of the intervention on Disability outcome (ODI) is significant				
1.0	BOOST		Walking Capacity	Disability (ODI)
If the indirect effect through walking capacity is significant from model 1.0				
1.1	BOOST	Physical Function (SPPB)	Walking Capacity	Disability (ODI)
1.2	BOOST	Physical Activity (RADI)	Walking Capacity	Disability (ODI)
1.3	BOOST	Fear avoidance (FABQ)	Walking Capacity	Disability (ODI)
1.4	BOOST	Self Efficacy Walking (SE-W)	Walking Capacity	Disability (ODI)
1.5	BOOST	Symptom Severity (SSS)	Walking Capacity	Disability (ODI)
If the indirect effect through walking capacity is not significant from model 1.0				
1.6	BOOST	Physical Function (SPPB)		Disability (ODI)
1.7	BOOST	Physical Activity (RADI)		Disability (ODI)
1.8	BOOST	Fear avoidance (FABQ)		Disability (ODI)
1.9	BOOST	Self Efficacy Walking (SE-W)		Disability (ODI)
1.10	BOOST	Symptom Severity (SSS)		
If the total effect of the intervention on Disability outcome (ODI) is not significant				
2.0	BOOST		Walking Capacity	Disability (ODI)
2.1	BOOST	Physical Function (SPPB)		Disability (ODI)
2.2	BOOST	Physical Activity (RADI)		Disability (ODI)
2.3	BOOST	Fear avoidance (FABQ)		Disability (ODI)
2.4	BOOST	Self Efficacy Walking (SE-W)		Disability (ODI)
2.5	BOOST	Symptom Severity (SSS)		Disability (ODI)

Multiple mediator models will only be tested if there is a significant relationship between M1 and M2. If the relationship is non-significant, then the alternative mediators will be tested in separate single mediator models. Significance levels are set a priori at p<0.05.
ODI = Oswestry Disability Index

For peer review only - http://bmjopen.bmj.com/site/about/guidelines.xhtml

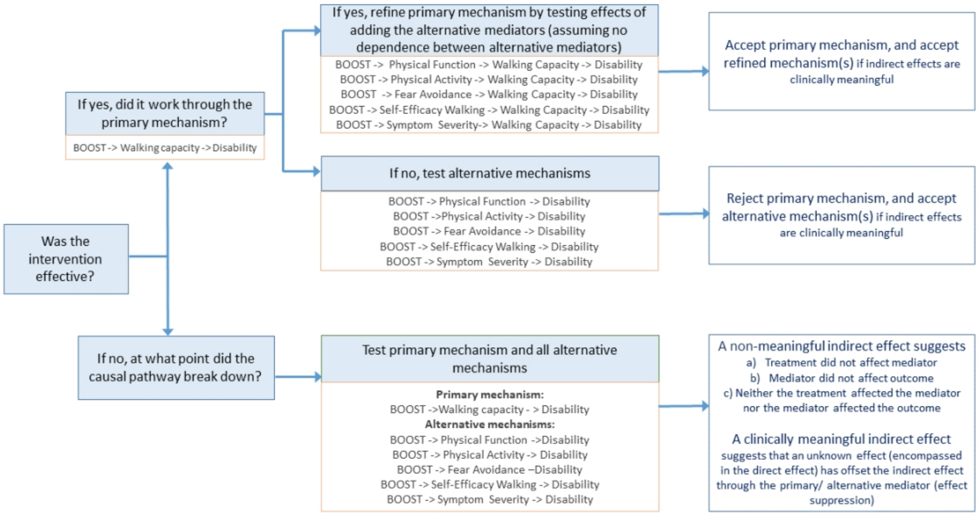

Heading for Figure 1:
Figure 1 Mediation protocol decision process

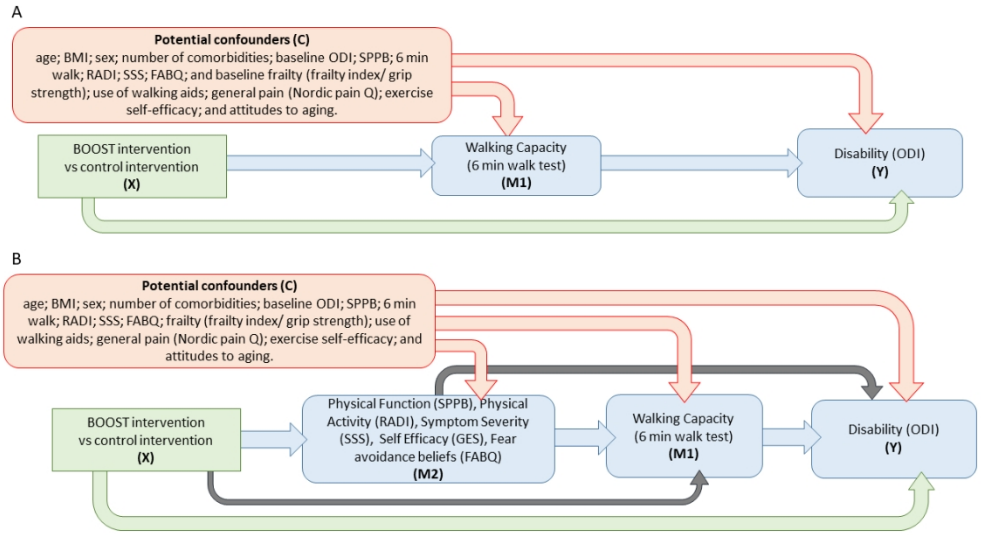

Heading for Figure 2: Figure 2 Figure 2 Directed acyclic graphics (DAG)

Legend for Figure 2: Blue lines represent indirect effects (mechanisms) of interest. Green lines represent direct effects (direct effect of treatment on outcome plus all unspecified indirect effects). Red lines represent possible effects that could induce confounding for indirect and direct effects. (A) A single mediator model where the intervention (X) exerts its effect on the outcome(s) Y, via an indirect path through the primary mediator (M1). (B) A serial multiple mediator model where the intervention (X) exerts its effect on the outcome (Y) via an indirect path through one of five alternative mediators (M2) and the primary mediator (M1), and a direct path (X to Y). ODI = Oswestry Disability Index, SPPB = S physical battery, RADI Rapid Assessment Disuse index, SSS = Swiss Spinal Stenosis Symptom severity scale, FABQ = fear avoidance beliefs questionnaire. M1 = 12 month measure, M2 = 6 month measures, Y = 12 month measure.
